# Thermal Decomposition of Molybdenum(II) Acetate as a Controlled Route to MoO_2_ and Mo_2_C Nanostructures: Structural, Morphological, and Atomic-Scale Insights

**DOI:** 10.1021/acsomega.6c04468

**Published:** 2026-08-17

**Authors:** Katiusse Soares de Souza, Crystian W. C. Silva, Anastasia Burimova, Levy Scalise, Gabriel Cabrera-Pasca, Juliana Schell, Larissa Otubo, Artur Wilson Carbonari

**Affiliations:** † Instituto de Pesquisas Energéticas e Nucleares, IPEN-CNEN/SP, São Paulo 05508-000, São Paulo, Brazil; ‡ Universidade Federal do Pará, Abaetetuba 09210-580, Pará, Brazil; § 30531 European Organization for Nuclear Research (CERN), Geneva CH-1211, Switzerland; ∥ Institute for Materials Science and Center for Nanointegration Duisburg-Essen (CENIDE), University of Duisburg-Essen, Essen 45141, Germany; ⊥ European Organization for Nuclear Research (CERN), Esplanade des Particules 1, Meyrin 1217, Switzerland

## Abstract

Developing reproducible synthetic routes for reduced molybdenum-based nanostructures remains challenging due to the strong sensitivity of Mo–O systems to temperature, processing atmosphere, and precursor chemistry. Here, we demonstrate that the controlled thermal decomposition of molybdenum­(II) acetate under vacuum provides a simple solid-state route for obtaining monoclinic MoO_2_ nanoparticles and hexagonal β-Mo_2_C with tunable morphology. Structural, morphological, and thermal analyses revealed a three-stage transformation pathway: (i) precursor degradation and amorphization between 300 and 400 °C; (ii) nucleation and crystallization of monoclinic MoO_2_ between 500 and 700 °C, yielding spherical nanoparticles with an average diameter of 23 ± 7.9 nm; and (iii) formation of vertically oriented MoO_2_/β-Mo_2_C microflakes at 800 °C, evidencing nanoparticle coalescence and the partial conversion of MoO_2_ into the β-Mo_2_C phase. Time-Differential Perturbed Angular Correlation (TDPAC) spectroscopy, using the ^111^In­(^111^Cd) probe, provided atomic-scale information on local electric field gradients, enabling the direct identification of amorphous and crystalline MoO_2_ environments while revealing the role of defects, oxygen vacancies, and surface heterogeneities during phase evolution. These findings establish molybdenum­(II) acetate as an effective precursor for the synthesis of molybdenum-based nanostructures and provide a multiscale understanding, spanning atomic to micrometer length scales, of the mechanisms governing the formation of MoO_2_ and β-Mo_2_C. Furthermore, the results indicate that carbonaceous species generated during precursor decomposition promote a coupled carbothermal reduction–carburization pathway, leading to the formation of β-Mo_2_C.

## Graphical abstract

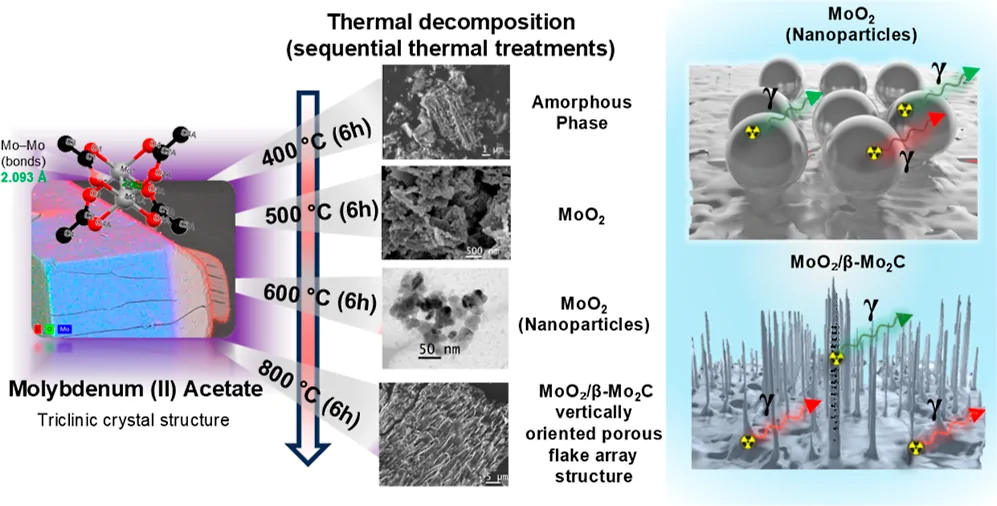

## 1 Introduction

The development of reproducible synthetic routes for molybdenum-based nanomaterials, particularly molybdenum oxides and carbides, is of growing interest due to their versatile structural, electronic, optical, and catalytic properties, which make them highly attractive for applications in catalysis, electrocatalysis, plasmonics, energy conversion and storage, sensing technologies, and conductive nanostructured materials.^1–4^ Among these materials, molybdenum dioxide (MoO_2_) has emerged as a particularly interesting system because of its unique combination of physicochemical, structural, electronic, optical, and catalytic properties.

Notably, MoO_2_ exhibits high chemical stability, strong electronic correlation effects, tunable defect chemistry, and superior thermal resistance compared with other semiconducting molybdenum oxide phases.^4–8^ Furthermore, its high electrical conductivity, arising from the significant contribution of Mo 3d electronic states near the Fermi level, endows MoO_2_ with metallic-like behavior.^9^ As a result, this compound has emerged as a promising candidate for applications in energy storage and conversion devices, sensors, electrodes, and advanced catalytic systems.^10–14^

In addition to its direct applications, MoO_2_ plays a crucial role as a precursor for the synthesis of molybdenum carbides, particularly the hexagonal β-Mo_2_C phase.^15–17^ This molybdenum carbide has attracted considerable interest owing to its noble metal-like catalytic behavior, exhibiting catalytic activities comparable to those of Pt and Pd.^1,2,18^ Consequently, β-Mo_2_C has emerged as a promising material for the hydrogen evolution reaction (HER), oxygen evolution reaction (OER), and a wide range of other electrocatalytic processes.^1,2,18–21^ This performance is generally attributed to the strong hybridization between Mo d and C p orbitals, which gives rise to an electronic structure that facilitates the adsorption and activation of reactive species. These characteristics, combined with high electrical conductivity, excellent thermal stability, and outstanding chemical resistance, make β-Mo_2_C a promising and cost-effective alternative to noble metal-based catalysts, expanding its potential for applications in energy conversion and storage.^2,22^

Several synthetic strategies have been developed for both the preparation of MoO_2_ and its subsequent conversion into β-Mo_2_C. Conventional approaches for the synthesis of MoO_2_ generally involve hydrothermal or solvothermal processes conducted under high-pressure conditions,^8,13,23^ multistep reduction routes starting from MoO_3_,^24,25^ or the use of external reducing atmospheres such as H_2_, CO, or forming gas.^26,27^ In turn, the conversion of MoO_2_ into β-Mo_2_C is typically achieved through carbothermal reduction, carburization in hydrocarbon-containing atmospheres, or chemical vapor deposition (CVD).^19,28^

In general, these routes involve the thermal treatment of MoO_2_ in the presence of carbon sources such as methane (CH_4_), acetylene (C_2_H_2_), ethane (C_2_H_6_), butane (C_4_H_10_), carbon monoxide (CO), acetone (C_3_H_6_O), or solid carbonaceous materials, including amorphous carbon, graphite, and carbon nanotubes.^1,2,16,19,29^ Under these conditions, the progressive incorporation of carbon into the molybdenum structure promotes the formation of the β-Mo_2_C phase. Furthermore, partial carburization of MoO_2_ may lead to the formation of hybrid MoO_2_/β-Mo_2_C heterostructures. In these heterostructures, the high electrical conductivity of MoO_2_ can act synergistically with the catalytic properties of β-Mo_2_C. As a result, hybrid MoO_2_/β-Mo_2_C materials often exhibit superior performance compared to the individual phases in electrocatalytic applications.^19,30–32^

Although highly effective, these synthetic approaches often require rigorous control of reaction conditions, specialized atmospheres, long processing times, or multiple synthetic steps to obtain MoO_2_, β-Mo_2_C, or hybrid MoO_2_ and β-Mo_2_C heterostructures. In addition to the challenges associated with synthetic control, recent studies have demonstrated that both the formation and functional performance of materials based on MoO_2_ and β-Mo_2_C depend not only on their crystal structure and morphology but also on local structural features such as defects, oxygen vacancies, interface density, atomic disorder, and the coexistence of amorphous and crystalline domains.^17,24–27,33^ In nanostructured systems, these factors can significantly modify the electronic structure, charge carrier mobility, and surface reactivity.^22,34–36^

Therefore, synthetic routes capable of controlling the structural and morphological evolution of molybdenum-based compounds, including the formation of intermediate phases and hybrid heterostructures, are of considerable interest for the development of advanced functional materials. Solid-state thermal decomposition has emerged as a particularly attractive approach for the synthesis of such materials. This method is based on the controlled thermal degradation of carboxylate precursors, followed by nucleation, crystal growth, and structural reorganization processes that ultimately lead to the formation of the final phases. This strategy has been successfully employed for the preparation of metallic nanostructures and oxides from acetate precursors of various transition metals, including Ag, Au, Cu, and Ni.^37–40^

Despite these advances, studies addressing the solid-state thermal decomposition of molybdenum precursors remain relatively scarce. In this context, Mo-based organometallic precursors containing preexisting Mo–Mo bonds and carbon-containing ligands represent particularly interesting systems, as they may provide intrinsically reducing decomposition pathways capable of promoting the formation of reduced molybdenum oxide phases, defect generation, and carbide formation during thermal treatment.^41–43^

A notable example is molybdenum­(II) acetate, also known as dimeric molybdenum acetate or tetrakis­(μ_2_-acetato)­dimolybdenum, [Mo_2_(CH_3_COO)_4_]. This binuclear organometallic complex exhibits strong metal–metal interactions and crystallizes in several polymorphic forms. The orthorhombic phase (α-Mo_2_(O_2_CCH_3_)_4_) is characterized by lattice parameters of *a* = 12.28 Å, *b* = 12.93 Å, and *c* = 5.50 Å, whereas two monoclinic variants have been reported with lattice parameters of *a* = 5.48 Å, *b* = 12.36 Å, and *c* = 19.86 Å, and *a* = 7.93 Å, *b* = 11.19 Å, and *c* = 5.57 Å. However, the triclinic structure is the most frequently reported polymorph, exhibiting lattice parameters of *a* = 8.41 Å, *b* = 5.50 Å, and *c* = 7.52 Å together with unique interaxial angles.^41,44,45^

As a member of a unique class of binuclear transition-metal complexes,^42,46^ the structural and electronic properties of molybdenum­(II) acetate are dominated by an exceptionally strong metal–metal bond. The short Mo–Mo distance of approximately 2.08 Å gives rise to significant electronic interactions and predominantly antiferromagnetic behavior.^43^ According to molecular orbital theory, this interaction is described as a quadruple bond consisting of one σ bond, two π bonds, and one δ bond.^44^ These structural and electronic characteristics impart unusually high thermal stability compared with other binuclear transition-metal acetates, such as those of Cr and Cu.^43,44,46^ Furthermore, during thermal decomposition, ligand fragmentation may generate locally reducing environments, while the partial preservation of short-range Mo–Mo correlations may influence nucleation, crystallization, and phase transformation processes. These features make molybdenum­(II) acetate a particularly attractive model system for investigating the interplay between amorphization, recrystallization, defect evolution, reduction, and carburization during the formation of MoO_2_ nanostructures, β-Mo_2_C, and related heterostructures through solid-state thermal decomposition. Consequently, a detailed understanding of the structural and morphological changes, as well as the local environments that emerge during this process, is essential, particularly considering that crystallization processes in these systems are often defect-mediated. Previous studies have mainly focused on the final crystalline products, neglecting the intermediate stages that involve amorphous phase formation, local structural disorder, oxygen-deficient environments, and nanoscale heterogeneity. Additionally, traditional structural techniques like X-ray diffraction (XRD) are limited in their ability to probe long-range crystalline order and may not accurately capture changes in local symmetry, interfacial disorder, or transient nonequilibrium environments that occur during decomposition.

Hyperfine techniques such as Time-Differential Perturbed Angular Correlation (TDPAC) spectroscopy provide a unique opportunity to investigate local atomic-scale environments during phase evolution. TDPAC is highly sensitive to electric field gradients surrounding radioactive probe nuclei and can therefore probe subtle variations in local symmetry, defect distribution, amorphous/crystalline coexistence, and atomic rearrangements that are frequently inaccessible to conventional diffraction methods.^47–51^ When combined with structural and morphological characterization techniques, TDPAC enables a multiscale understanding of the crystallization and reduction processes governing the formation of Mo-based nanostructures.

To the best of our knowledge, no previous study has systematically correlated the thermal decomposition of molybdenum­(II) acetate with the simultaneous evolution of the crystal structure, morphology, and atomic-scale hyperfine environments over a broad temperature range. While previous investigations have largely focused on the synthesis of either MoO_2_ or Mo_2_C using multistep reduction or carburization routes, the present work demonstrates that both phases can emerge from a single-source organometallic precursor through a controlled vacuum-assisted decomposition process. Furthermore, the combination of TDPAC spectroscopy with conventional structural characterization provides unique insights into the local ordering processes that precede crystallization, revealing transformation stages that cannot be detected by diffraction techniques alone.

In this study, we investigate the vacuum-assisted thermal decomposition of molybdenum­(II) acetate as a solid-state route for the synthesis of MoO_2_ nanoparticles and hybrid MoO_2_/β-Mo_2_C microstructures. Particular emphasis is placed on elucidating the structural and microstructural mechanisms governing phase evolution during thermal treatment, including amorphization, crystallization, defect formation, reduction, and carburization processes. By correlating phase evolution, morphological transformations, and local atomic-scale environments through XRD, SEM/HRTEM, thermal analysis, and TDPAC spectroscopy, this work provides a comprehensive understanding of defect-mediated structural evolution during precursor decomposition and the mechanisms controlling the formation of functional molybdenum oxide and carbide nanomaterials. Scheme 1 presents a schematic illustration of the thermal decomposition of molybdenum­(II) acetate under vacuum, as well as the characterization strategies employed to investigate the structural evolution from the molybdenum­(II) acetate precursor to the formation of the crystalline MoO_2_ and Mo_2_C phases.

**Scheme 1:**
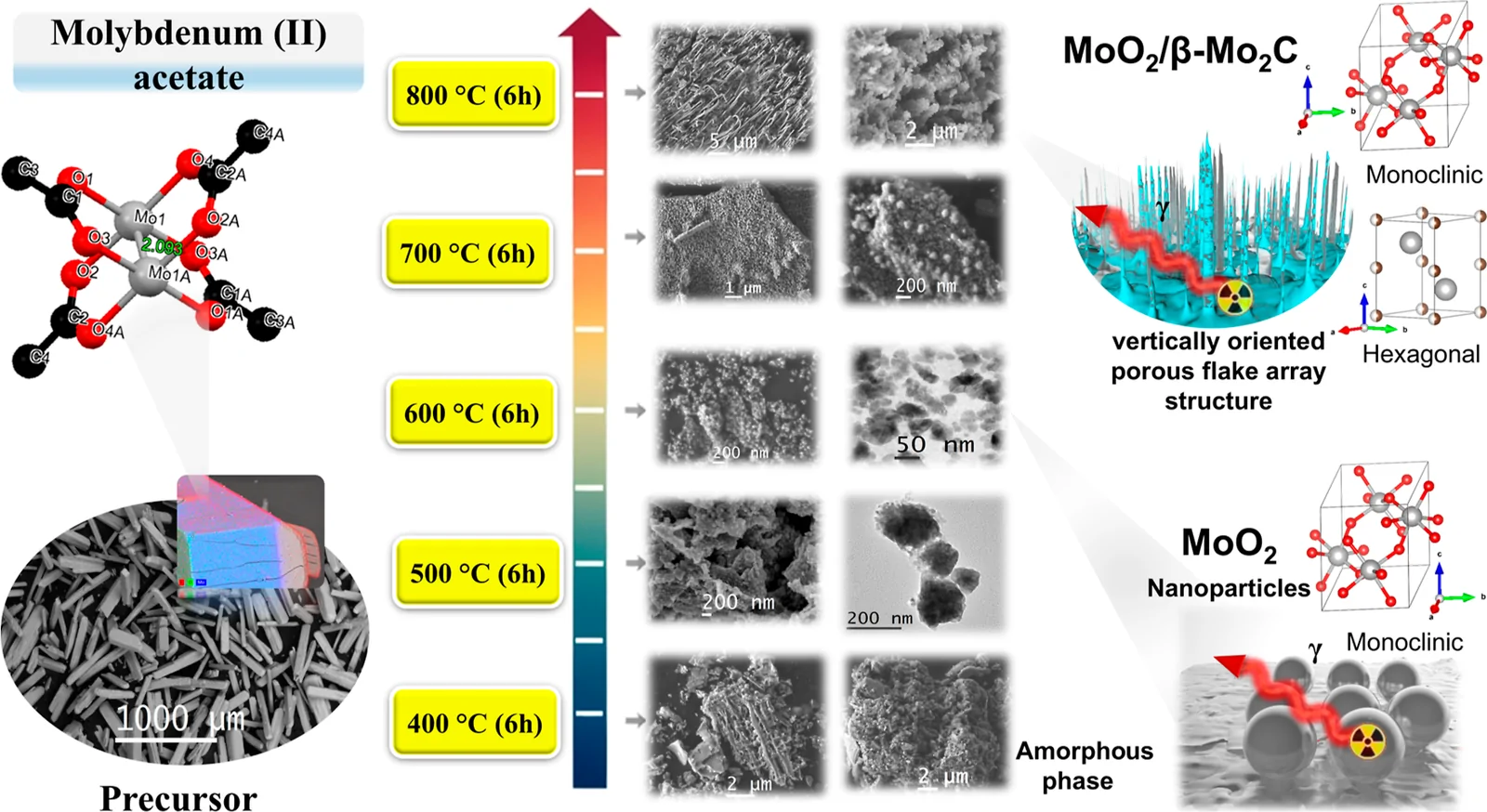
Schematic Representation of the Synthesis and Characterization of MoO_2_ Nanoparticles from Molybdenum­(II) Acetate

## 2 Results and Discussion

### 2.1 Characterization of Molybdenum­(II) Acetate at Room Temperature

Figure 1a presents the X-ray diffraction pattern of molybdenum­(II) acetate, indicating a triclinic crystal structure belonging to the *P*1 space group, with preferred orientation along the (001) plane and a relative crystallinity of approximately 95%. The observed preferred orientation can be attributed to the intrinsic structural features of the compound. Molybdenum­(II) acetate forms centrosymmetric dimers of the type Mo_2_(OAc)_4_, in which two molybdenum atoms are connected by a short metal–metal bond and stabilized by four acetate groups in a bridging configuration. Each metal center exhibits an approximately distorted square-pyramidal coordination environment, in which the Mo–Mo bond occupies the axial position, while the oxygen atoms from the acetate groups define a plane around the metal centers. This structural arrangement imparts directionality to the molecular units, as the metal–metal bond defines a preferential axis within the dimer. When arranged in the crystalline lattice, these dimeric units interact anisotropically, favoring stronger interactions along specific crystallographic directions. As a result, crystal growth tends to occur preferentially along these directions, which may account for the preferred orientation observed along the (001) plane in the X-ray diffraction pattern. The crystallinity of the sample was estimated from the ratio between the areas corresponding to the crystalline and amorphous regions of the diffraction pattern by the Integration Method (also known as the Ruland–Vonk method).^52–55^ The estimation yields an approximate value of 95%, indicating a high degree of structural ordering.^33,56–58^ This is a relative rather than an absolute value.

**Figure 1:**
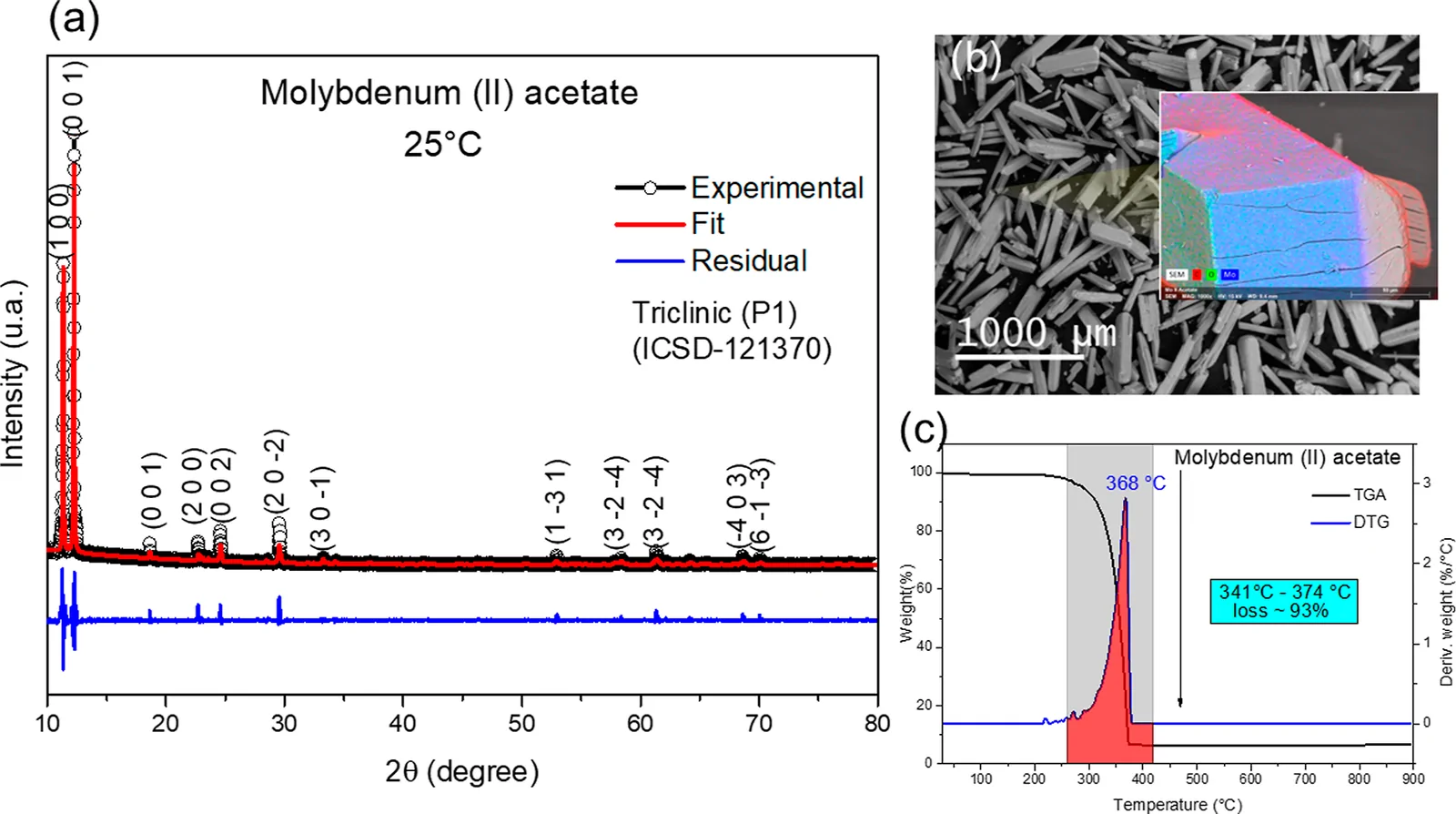
Structural, morphological, and thermal characterization of commercial molybdenum­(II) acetate. (a) XRD pattern (XRD), (b) scanning electron microscopy (SEM) and energy-dispersive spectroscopy (EDS) spectrum, and (c) thermogravimetric analysis (TGA) curve.

The morphology of the sample is presented in Figure 1b. Scanning electron microscopy (SEM) micrographs indicate that the solid is predominantly composed of elongated microstructures with rodlike morphology. This morphological feature is consistent with the preferred orientation observed in the X-ray diffraction (XRD) results. Furthermore, elemental analysis by energy-dispersive spectroscopy (EDS) confirms the presence of molybdenum (Mo), oxygen (O), and carbon (C), which are the constituent elements of acetate.^33^

The results of the thermal characterization by thermogravimetric analysis (TGA), presented in Figure 1c, indicate that under an inert atmosphere (nitrogen), molybdenum­(II) acetate remains thermally stable up to approximately 300 °C. Above this temperature, a pronounced decomposition event is observed, characterized by a significant mass loss in the range between 341 and 374 °C, corresponding to approximately 93% of the initial mass. This process is associated with the decomposition of the organic acetate ligands and the release of volatile species, resulting in the formation of inorganic molybdenum species. The occurrence of a single, well-defined decomposition event suggests a relatively homogeneous thermal process, consistent with the high degree of crystallinity observed for the precursor material.^34,48,59^

### 2.2 Characterization of Molybdenum­(II) Acetate in the Temperature Range from 300 to 800 °C

To understand the structural changes associated with the thermal phenomena observed in molybdenum acetate, heat treatments were carried out in the temperature range from 300 to 800 °C, in increments of 100 °C, with a duration of 6 h at each step, under a vacuum atmosphere.

The results of the structural and morphological characterizations at 300 °C are presented in Figure 2a,b. The XRD result shown in Figure 2a indicates that the diffraction peaks characteristic of the triclinic structure of molybdenum­(II) acetate (ICSD-1213070), space group *P1*, are still present, suggesting partial preservation of the precursor after thermal treatment. However, a loss of preferential orientation along the (001) plane is observed in comparison with the initial pattern (Figure 1a), as well as a significant reduction in relative crystallinity, from approximately 95% to 10%. This result is corroborated by the morphological and elemental characterization presented in Figure 2b, which shows that, after thermal treatment, the sample does not exhibit a specific morphology (rod-like). Taken together, these results indicate a significant loss of long-range crystalline order, possibly associated with the onset of thermal decomposition of the coordinated acetate groups,^26^ characterizing a process of partial structural degradation of the precursor under the employed conditions.

**Figure 2:**
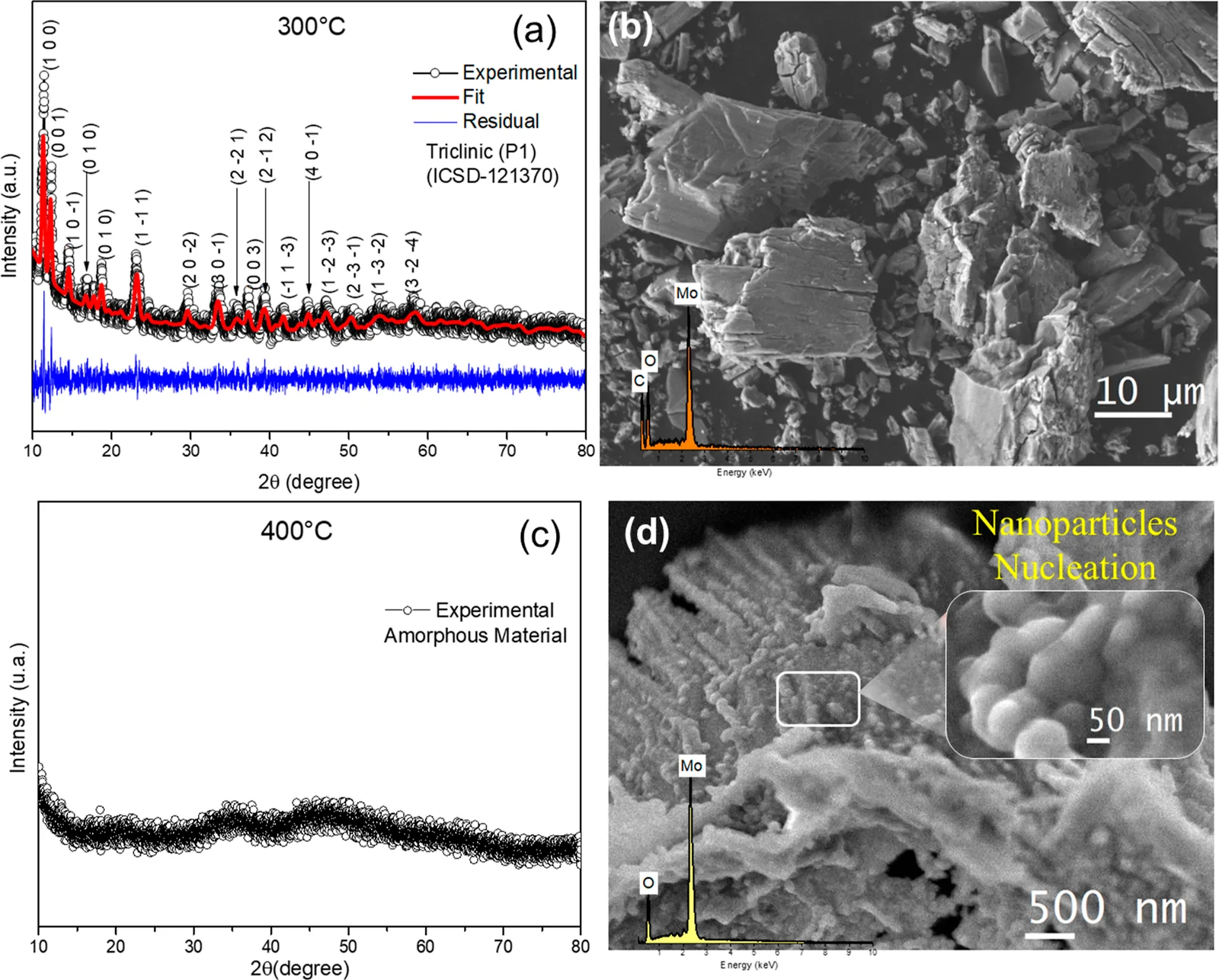
Structural and morphological results of molybdenum acetate at 300 and 400 °C: (a) XRD obtained after thermal treatment at 300 °C; (b) SEM/EDS obtained after thermal treatment at 300 °C; (c) XRD obtained after thermal treatment at 400 °C; and (d) SEM/EDS obtained after thermal treatment at 400 °C.

The results obtained at 400 °C are presented in Figure 2c,d. The observed diffraction pattern is characteristic of an amorphous material, evidencing the loss of long-range structural order. Although the material appears amorphous from the perspective of long-range structural order, the decomposition process is unlikely to produce a completely random atomic arrangement. Instead, the resulting phase probably consists of short-range Mo–O and residual Mo–Mo correlated domains inherited from the precursor structure. Such short-range ordering may act as nucleation centers for the subsequent crystallization of MoO_2_. This interpretation is consistent with the later TDPAC results, which reveal the emergence of locally ordered environments before the appearance of well-defined diffraction peaks.

This behavior is also consistent with the significant mass loss observed around 370 °C in the thermal analysis, as well as with results reported in the literature, which associate this temperature range with the decomposition of acetate groups, accompanied by the release of volatile species such as acetone (CH_3_COCH_3_), carbon dioxide (CO_2_), methane (CH_4_), and other light carbonaceous compounds.^34,48,59^

Considering the initial triclinic structure of molybdenum­(II) acetate, characterized by bridging acetate groups and short, ordered intermetallic bonds, the increase in temperature promotes the progressive rupture of these interactions. The release of volatile species contributes to the structural disordering of the crystal lattice, resulting in the transition to an amorphous state, as experimentally observed.^41,42^ Additionally, these changes exert a direct influence on the morphology of the material, as evidenced in the micrograph presented in Figure 2d, which reveals indications of nanoparticle nucleation on the surface regions of the sample at 400 °C. This behavior is consistent with that observed for other transition metal acetates, such as Cu, Ni, and Ag, in which thermal decomposition induces the formation of metallic or oxide nanometric nuclei.^37,60^ Moreover, the release of these volatile species may act as an auxiliary reduction mechanism during thermal evolution, creating a locally partially reducing environment. This effect may hinder the formation of phases with higher oxidation states, such as MoO_3_, favoring, in subsequent stages, the stabilization of reduced phases such as MoO_2_.^34^

The crystallization of the monoclinic phase of molybdenum dioxide (MoO_2_), space group *P*2_1_/*c* (ICSD-644064), is confirmed from 500 °C, as shown in Figure 3a. At this temperature, the increase in thermal mobility favors structural reorganization and the formation of MoO_6_ octahedral units, which arrange into chains characteristic of the monoclinic MoO_2_ phase.^4,61^ As previously observed, thermal treatment under vacuum, combined with the partially reducing atmosphere generated by the decomposition of the acetate precursor, plays a fundamental role in this process. This environment limits oxygen availability, favoring the stabilization of the +4 oxidation state of molybdenum, inhibiting the formation of MoO_3_.^5,62^

**Figure 3:**
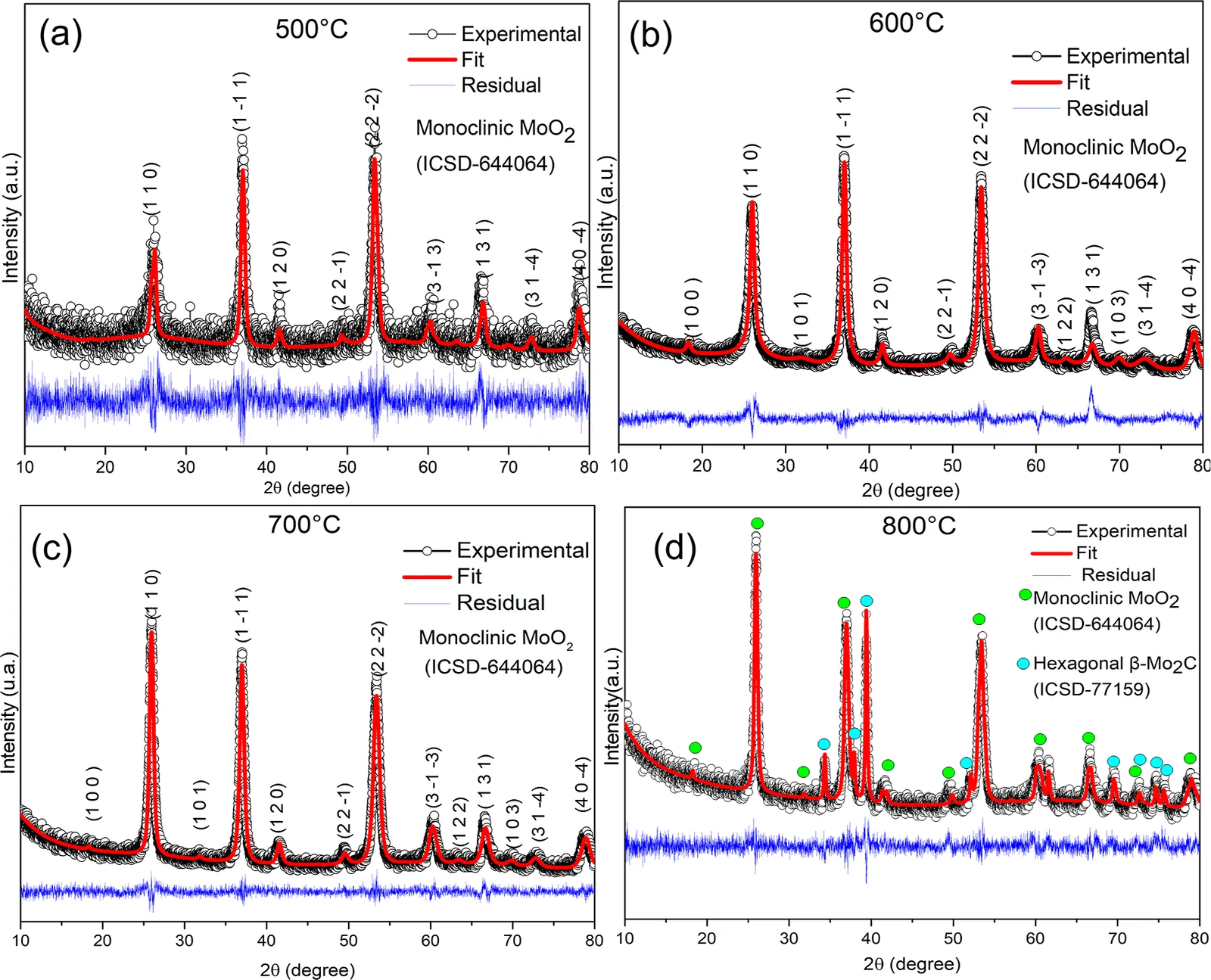
XRD results obtained as a function of temperature: (a) XRD obtained after thermal treatment at 500 °C; (b) XRD obtained after thermal treatment at 600 °C; (c) XRD obtained after thermal treatment at 700 °C; and (d) XRD obtained after thermal treatment at 800 °C.

From a chemical perspective, the formation of MoO_2_ is likely governed by a self-reduction process induced by carbonaceous decomposition products generated during the acetate degradation. The release of CO, hydrocarbons, and other reducing species may locally decrease the oxygen chemical potential, thereby stabilizing Mo^4+^ instead of higher oxidation states, such as Mo^6+^. Consequently, the precursor behaves not only as a molybdenum source but also as an intrinsic reducing agent, eliminating the need for external hydrogen-containing atmospheres that are commonly employed in MoO_2_ synthesis.

Furthermore, Figure 3b,c demonstrates the stability of the monoclinic MoO_2_ phase in the temperature range between 500 and 700 °C. No additional crystalline phases were detected, and only changes in the degree of crystallinity were observed. The sample treated at 500 °C exhibited a crystallinity of approximately 75%, which progressively increased to 82% and 86% after thermal treatments at 600 and 700 °C, respectively. These results indicate a progressive increase in structural ordering while preserving the phase composition throughout this temperature interval.

This behavior shows a qualitative correlation with the differential scanning calorimetry (DSC) results obtained for molybdenum­(II) acetate under a nitrogen atmosphere (see Figure S1a in the Supporting Information). Under these conditions, an intense exothermic event is observed between approximately 500 and 700 °C, indicating the occurrence of structural reorganization processes and an increase in crystalline order. Although the DSC experiments were performed under an N_2_ atmosphere and the thermal treatments for X-ray diffraction were carried out under vacuum, the coincidence between the temperature range of the exothermic event and the progressive increase in the crystallinity of MoO_2_ suggests that both results are associated with similar structural transformations occurring after the decomposition of the precursor.^63^ It is important to stress that TGA/DSC under N_2_ atmosphere measurements provide only an approximate thermal reference for the vacuum-sealed synthesis conditions because gas transport, pressure evolution, and oxygen partial pressure differ between both environments, and therefore, the mechanistic interpretation must be treated cautiously.

After heat treatment at 800 °C, a structural phase transition is observed, as shown in Figure 3d. Rietveld refinement reveals the coexistence of two crystalline phases: monoclinic MoO_2_, corresponding to approximately 84 wt %, and β-Mo_2_C (ICSD-77159), present as a secondary phase with about 16 wt %. The estimated relative crystallinity for this sample was approximately 90%.

The formation of β-Mo_2_C indicates the occurrence of partial carbothermal reduction under vacuum conditions. Residual carbonaceous species generated during the decomposition of the acetate precursor act as carbon sources, favoring the partial conversion of MoO_2_ into β-Mo_2_C. Similar transformation pathways have been reported for molybdenum-based precursors treated under reducing atmospheres, in which carbon released during precursor decomposition contributes to carbide formation at elevated temperatures.^1,2,19^

The relatively low fraction of β-Mo_2_C (≈16 wt %) suggests that carbon availability becomes the limiting factor during the carburization stage. Under the present vacuum conditions, a substantial portion of the carbon generated during acetate decomposition is likely removed as volatile species at lower temperatures, leaving only a fraction available for carbide formation at 800 °C. Therefore, the final phase composition appears to be controlled by a competition between oxygen removal and carbon retention during thermal evolution.

These observations are in qualitative agreement with the thermal analysis (TGA/DSC) performed on the MoO_2_ sample previously obtained at 500 °C (see Figure S1b in the Supporting Information). The TGA/DTG curves reveal a mass-loss event beginning at approximately 755 °C, accompanied by an endothermic DSC event centered at approximately 768 °C. Although these measurements were performed under a nitrogen atmosphere and the X-ray diffraction analysis conducted after the thermal experiments identified only a small fraction of bcc-Mo (approximately 3 wt.%; see Figure S1c in the Supporting Information), the results suggest that oxygen loss in the vacuum-treated samples occurs within a similar temperature range. Under these conditions, the formation of oxygen-deficient regions (MoO_2‑x_) may act as an intermediate step in the nucleation of β-Mo_2_C, while the limited availability of carbonaceous species favors the formation of a hybrid MoO_2_/β-Mo_2_C system (see Figure S2 in the Supporting Information).^1,64^

In view of the structural changes observed in the temperature range from 500 to 800 °C, the morphological evolution within this range was investigated using electron microscopy techniques (SEM/HRTEM). Figure 4 shows the results obtained at 500 and 600 °C, respectively. The results for the sample at 500 °C (Figure 4a–e) show the formation of “plates” decorated with nanoparticles; these plates exhibit heterogeneous shapes. TEM micrographs show that, in this system, both the regions of particle agglomerates (approximately 170 nm) and the interfacial regions of these agglomerates exhibit crystalline ordering. HRTEM images obtained from particle surfaces (Figure 4d,e) show the (110) and (020) crystallographic planes of the monoclinic MoO_2_ phase, in agreement with the crystallographic orientation along the [001] zone axis.

**Figure 4:**
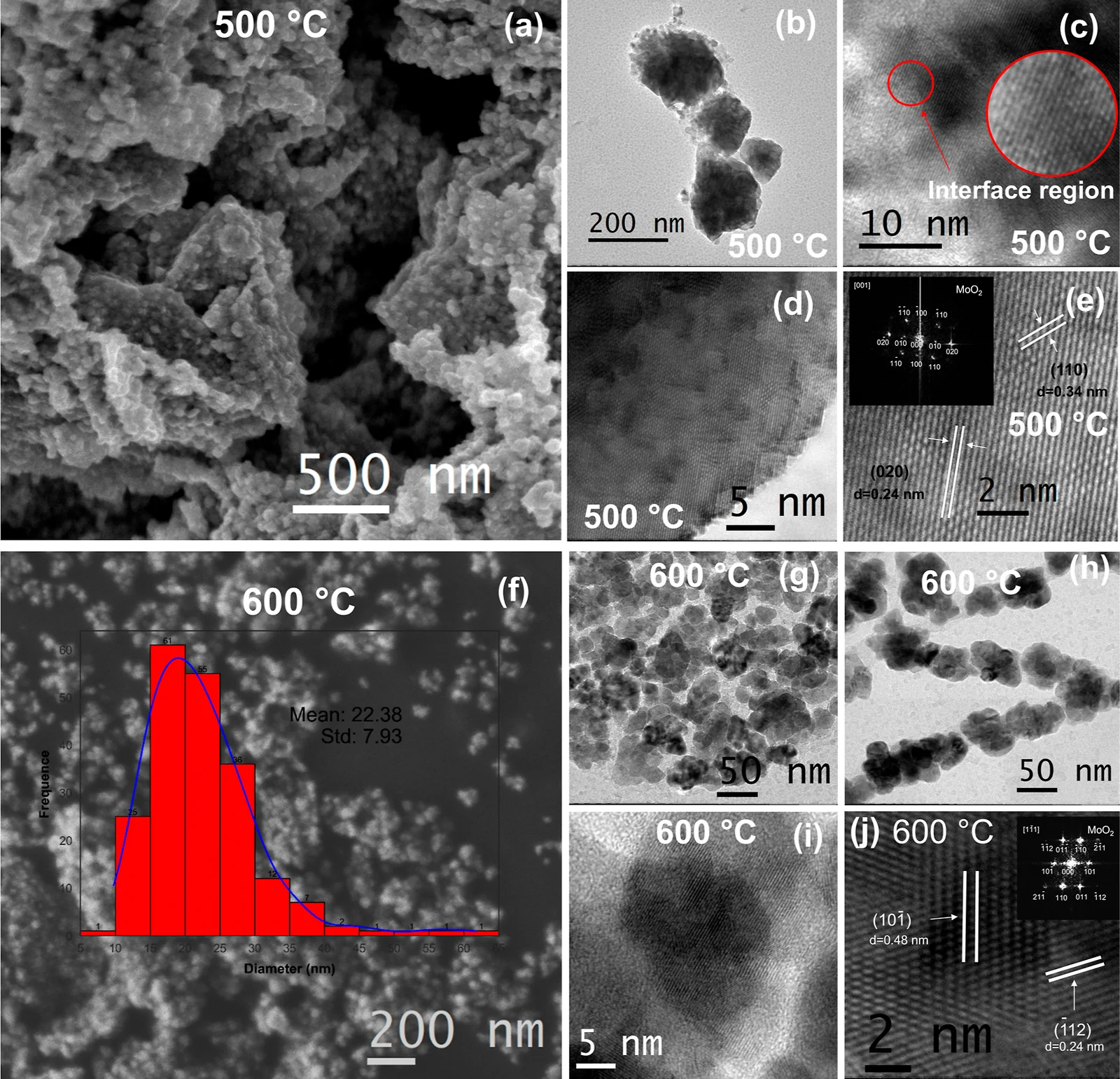
SEM, TEM, and HRTEM micrographs of samples treated at 500 and 600 °C. (a) SEM image of the sample treated at 500 °C ; (b) TEM image of particle agglomerates; (c) interfacial region between adjacent particles; (d,e) HRTEM images showing the crystallographic planes of monoclinic MoO_2_ along the [001] zone axis. (f) Representative image used for particle-size analysis at 600 °C; (g,h) TEM images of MoO_2_ nanoparticles; and (i,j) HRTEM images showing the crystallographic planes of monoclinic MoO_2_ along the [1 1̅ 1] zone axis.

Figure 4f–j corresponds to the results for the sample at 600 °C. The micrograph presented in Figure 4f shows the formation of dispersed nanoparticles at this stage, indicating the segmentation of the particle agglomerates observed previously. These results are confirmed by the TEM/HRTEM analyses shown in Figure 4g–j. The size distribution analysis for a population of 200 nanoparticles suggests an average diameter (*D*_*m*_) of approximately 22.4 nm, with a standard deviation of 7.9 nm (σ_*D*_m__ = ±7.9nm). Furthermore, hierarchical clustering analysis of the diameter distribution, using Ward’s method, reveals that the sample is composed of three statistically distinct populations, centered at approximately 18.3 nm (68%), 29.8 nm (30.5%), and 57.8 nm (1.5%) (see Figure S3 in the Supporting Information). Additionally, the crystalline nature of these nanoparticles is evidenced by the HRTEM analysis (Figure 4j), which reveals the (101) and (112) crystallographic planes of monoclinic MoO_2_, consistent with the [1 1̅ 1] zone axis. From this perspective, the formation of nanoparticles during thermal evolution is consistent with the behavior commonly reported for the solid-state thermal decomposition of transition-metal acetates. Similar morphological evolution has been reported for Ag, Cu, and Ni acetates, as well as for molybdenum oxide systems undergoing thermally induced phase transformations.^11,27,37–40,60^

Figure 5 shows the results of the morphological characterizations at 700 and 800 °C, respectively. At 700 °C, the SEM micrographs suggest the coalescence of nanoparticles and the formation of micrometric structures decorated with remnant nanoparticles on the surface.

**Figure 5:**
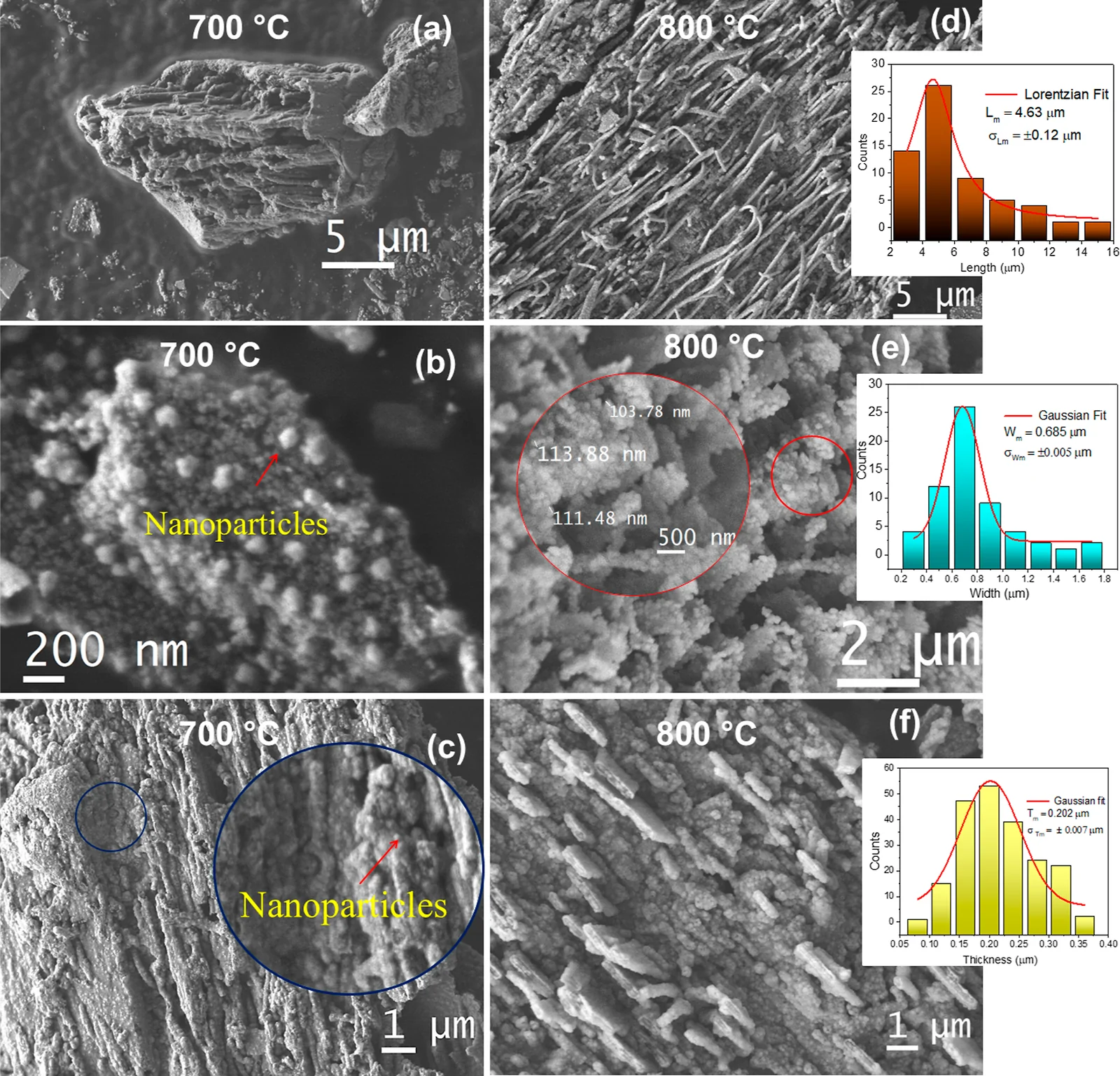
Morphological evolution of samples treated at 700 and 800 °C. (a) SEM image of the sample treated at 700 °C; (b) higher-magnification image highlighting the formation of nanoparticle agglomerates; (c) surface detail of the microstructure showing adhered nanoparticles. (d) SEM image of vertically oriented microflakes formed at 800 °C, together with their length distribution; (e) higher-magnification image showing nanoparticles adhered to the microflake surfaces and the corresponding width distribution; and (f) vertical view of the microflakes together with the corresponding thickness distribution.

At 800 °C, the micrographs indicate the formation of vertically oriented MoO_2_/β-Mo_2_C microflakes with an average length (*W*_*m*_) of 4.3 ± 0.1 μm (*n* = 60). Figure 5e,f corresponds to higher-magnification images of these structures and provides information on their width and thickness distributions. As shown in Figure 5e, the average width (*L*_*m*_) was 0.685 ± 0.005 μm (*n* = 60), whereas the average thickness (*T*_*m*_) was 0.202 ± 0.007 μm (*n* = 200), as presented in Figure 5f.

The oxygen-deficient conditions generated during vacuum treatment may also promote the formation of oxygen vacancies within the MoO_2_ lattice. Such defects have been widely reported to modify the electronic structure, conductivity, and catalytic activity of molybdenum oxides. Although the present work does not directly quantify vacancy concentrations, the combined structural and hyperfine results strongly suggest that oxygen-deficient local environments contribute to the observed disorder and may participate in the subsequent carburization process leading to β-Mo_2_C formation.

The morphological evolution observed between 700 and 800 °C suggests that the coalesced structures formed at lower temperatures (Figure 5a–c) act as structural templates for subsequent growth. Similar flake-like morphologies have been reported in MoO_2_-based systems, where the anisotropic nature of the monoclinic crystal structure promotes preferential growth along specific crystallographic directions, leading to the formation of lamellar and vertically oriented architectures.^13,27,65^ In this context, the increase in thermal energy at 800 °C enhances atomic diffusion and surface mobility, facilitating the reorganization of previously formed nanoparticles and coalesced domains into larger and more ordered structures.

Simultaneously, the structural transformations identified by X-ray diffraction indicate the onset of partial carburization, resulting in the formation of a hybrid MoO_2_/β-Mo_2_C system. The coexistence of these phases, together with the oxygen-deficient conditions imposed by the vacuum atmosphere, may contribute to local compositional gradients that further promote mass transport and recrystallization processes. Consequently, anisotropic crystal growth, diffusion-assisted restructuring, and partial carburization act synergistically to produce the vertically oriented microflake morphology observed at 800 °C.^13,27,65^ The presence of residual nanoparticles decorating the microflake surfaces suggests that the coalescence process is not yet complete, indicating a progressive morphological evolution.

### 2.3 Results of Hyperfine Interactions at the ^111^In­(^111^Cd) Radioactive Probe

To investigate the structural evolution from an atomic-scale perspective, radioactive ^111^In (^111^Cd) probe nuclei were thermally diffused into the samples at 300 °C for 6 h. The resulting materials were subsequently characterized by the Time-Differential Perturbed Angular Correlation (TDPAC) technique, with the aim of probing the hyperfine interactions occurring at each stage of thermal evolution. Since TDPAC probes the electric field gradient (EFG) at the probe nucleus, it is highly sensitive to local atomic environments, short-range ordering, and structural heterogeneity, complementing the information obtained from diffraction-based techniques.

It is important to emphasize at the outset the complementary natures of TDPAC and XRD. While XRD is sensitive to long-range crystalline order and provides macroscopic structural information averaged over the bulk of the sample, TDPAC is intrinsically sensitive to the local atomic environment surrounding the probe nucleus on a scale of a few angstroms. Consequently, TDPAC can detect local crystalline environments, defect distributions, and amorphous/crystalline coexistence even under conditions where XRD does not resolve well-defined Bragg reflections. This distinction is crucial for interpreting the results presented in the following.

The time-dependent anisotropy ratio functions (*R*(*t*)) and their corresponding Fourier transforms (FFT), which allow analysis in the frequency domain and aid in the identification of the hyperfine components, are shown in Figure 6. Measurements were performed at room temperature (25 °C) after probe diffusion and subsequently during thermal treatment at 400–800 °C. All TDPAC spectra were fitted by a model using a theoretical function considering electric quadrupole interactions only. Initially, after thermal diffusion, the spectrum exhibited one site with a broad frequency distribution, denoted site 1, indicating the presence of an environment without well-defined local symmetry. Additional sites (site 2 and site 3) emerge throughout the thermal evolution and are attributed to probe nuclei in more ordered, crystalline-like environments.^48,66^

**Figure 6:**
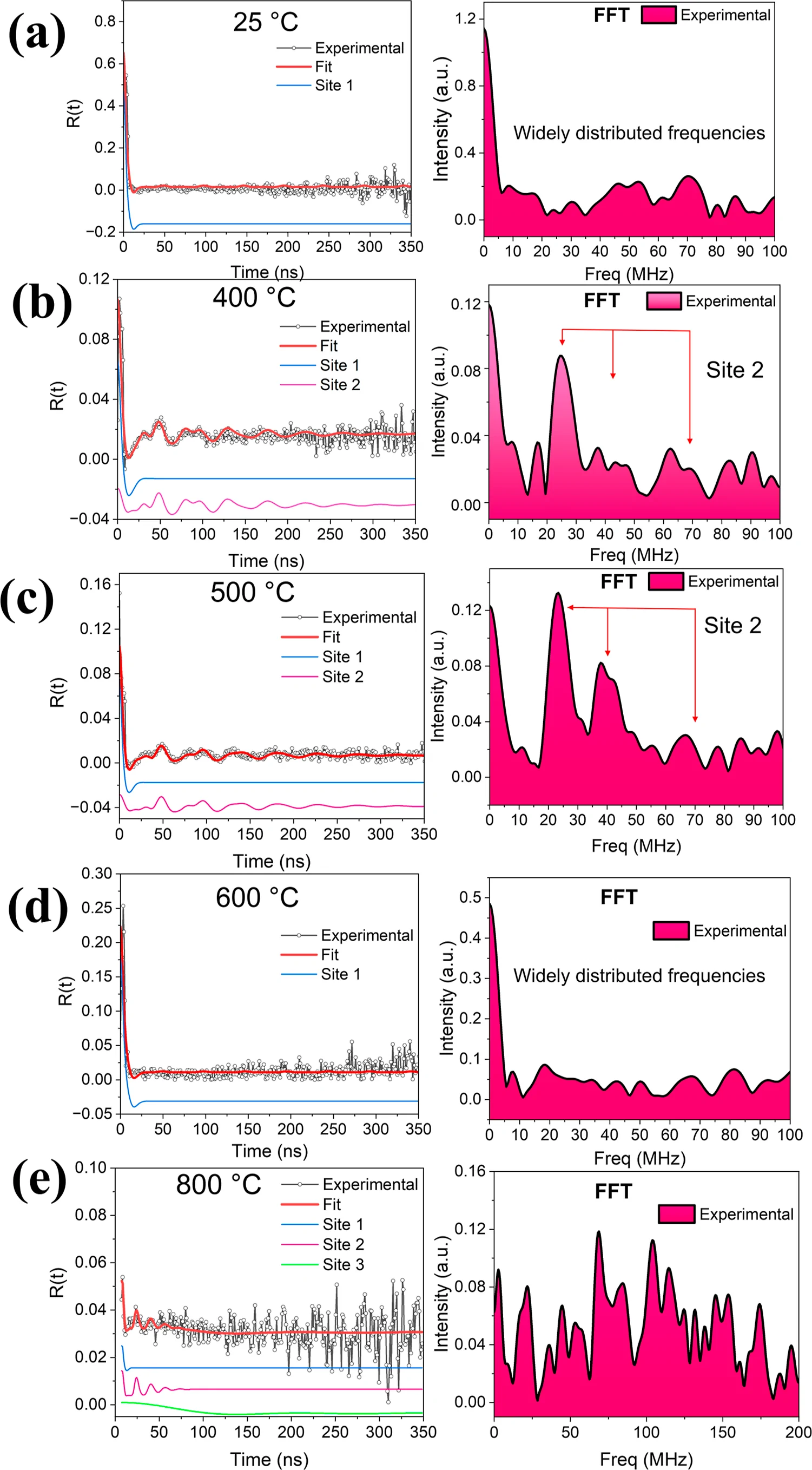
Time-dependent anisotropy ratio function (TDPAC) and their corresponding FFTs at the indicated temperatures: (a) 25 °C; (b) 400 °C; (c) 500 °C; (d) 600 °C; (e) 800 °C.

Several factors may account for the persistent presence of site 1 throughout the thermal evolution in the present system, which may suggest that a fraction of the probe nuclei remains associated with structurally heterogeneous regions generated during the precursor decomposition and nanoparticle growth. A plausible explanation is that a significant fraction of the probe nuclei resides on the surface of the sample, as previously reported by Effenberger et al.^66^ Surface enrichment leads to a high concentration of probes in heterogeneous local environments, contributing to the broad frequency distributions observed. Additionally, the presence of carboxylic groups in the precursor may increase the diffusion affinity of the probes toward surface sites, further enhancing spectral broadening. The temperature conditions during probe diffusion may also play a role: insufficient thermal energy may limit the incorporation of probe nuclei into substitutional bulk lattice sites, whereas excessively elevated temperatures may promote surface degradation and further disorder.

As shown in Figure 6a, the spectrum at 25 °C is characterized exclusively by site 1, with a widely distributed frequency pattern that reflects a highly heterogeneous local environment, indicating a significant structural disorder of the precursor after probe diffusion. No crystalline hyperfine environment could be resolved at this temperature. This is consistent with the severe degradation of the precursor crystallinity and the onset of structural disorder, already observed by XRD at 300 °C, where the relative crystallinity of molybdenum­(II) acetate was found to have decreased from approximately 95% to 10%.

A particularly important observation is obtained for the sample treated at 400 °C (see Figure 6b). Although XRD indicates predominantly amorphous behavior at this temperature, TDPAC detects a small fraction of probe nuclei occupying a well-defined local environment characterized by ν_*Q*_ ≈ 138 MHz and a relatively narrow frequency distribution. This apparent discrepancy is not contradictory but rather reflects the different structural length scales probed by the two techniques. XRD is sensitive to long-range crystallographic order, whereas TDPAC probes only the immediate atomic neighborhood of the radioactive nucleus. Consequently, local ordering can already be detected by TDPAC even when the crystalline domains remain too small, defective, or insufficiently connected to generate detectable diffraction peaks. Together with the SEM observations showing the onset of nanoparticle nucleation at 400 °C, these results suggest the formation of locally ordered regions during the amorphous-to-crystalline transition. This finding demonstrates that local crystalline ordering, likely associated with the early nucleation of MoO_2_ nanodomains, precedes the onset of long-range crystalline order detectable by XRD. This is physically consistent with the nucleation mechanism: short-range atomic rearrangements and the formation of incipient crystalline nuclei can produce well-defined local symmetries around the probe nucleus before these domains grow to sizes sufficient for coherent X-ray scattering.

This observation highlights one of the principal advantages of hyperfine techniques for studying phase transformations in nanomaterials. Whereas diffraction methods require coherent crystalline domains of sufficient size to generate measurable Bragg reflections, TDPAC remains sensitive to local atomic ordering over only a few nearest neighbor shells. Consequently, the technique can detect precursor stages of crystallization and nucleation events that occur before the establishment of long-range structural coherence.

Upon increasing the temperature to 500 °C (see Figure 6c), the same hyperfine interaction remains present with very similar parameters. At this temperature, XRD and HRTEM unequivocally demonstrate the formation of crystalline monoclinic MoO_2_ nanoparticles and would support the assignment of site 2 to a MoO_2_-like local environment. The persistence of this hyperfine environment therefore indicates that the local ordering detected at 400 °C evolves into the crystalline environments associated with the MoO_2_ phase. However, considering the absence of reference hyperfine parameters for ^111^Cd in MoO_2_, we refrain from assigning this interaction to a specific crystallographic site. The present results only demonstrate that this component corresponds to a well-defined locally ordered environment that emerges during MoO_2_ nucleation and remains stable during subsequent crystallization.

It should be stressed that the assignment of site 2 to a specific crystallographic environment within MoO_2_ remains tentative at this stage. The hyperfine parameters of ^111^In (^111^Cd) probes in MoO_2_ have not been systematically established in the literature. For comparison, hyperfine studies in related molybdenum oxide systems, particularly α-MoO_3_, have reported quadrupole frequencies in the range of 100–200 MHz depending on the oxygen vacancy configuration and local coordination geometry.^51^ The ν_*Q*_ value obtained here (138 MHz) falls within this range and is consistent with a Mo-oxide environment with partial local disorder, but a definitive crystallographic assignment requires further investigation, ideally supported by first-principles calculations of the electric field gradient tensor in MoO_2_ structures with and without defects.

At 600 °C (Figure 6d), the TDPAC spectrum no longer exhibits a well-defined frequency component and is instead characterized by a broad frequency distribution (δ = 90%), typical of probes in highly perturbed or surface-dominated environments. This result is particularly informative when interpreted in conjunction with the morphological characterization. Although XRD and HRTEM clearly confirm the presence of crystalline MoO_2_, SEM and TEM analyses reveal that the previously agglomerated nanoparticle assemblies have segmented into dispersed nanoparticles, with 68% of the observed population having diameters below approximately 20 nm.

Such nanostructuring dramatically increases the surface-to-volume ratio and defect-rich regions that accompany this size reduction and is directly reflected in the TDPAC signal. The predominance of the distributed hyperfine interaction therefore likely reflects the increasing influence of these heterogeneous local environments on the probe nuclei that were previously located at interfacial or grain boundary regions within larger agglomerates now predominantly reside at the surfaces of the smaller, segmented nanoparticles, where the local environment is highly heterogeneous and disordered. This interpretation is consistent with previous TDPAC studies in nanoparticle systems, where surface environments dominate the measured hyperfine response despite the presence of long-range crystallinity, and that demonstrated a strong correlation between particle size reduction and the broadening of hyperfine frequency distributions.^48,49,66^ The same general behavior remains observable at 700 °C (see Figure S4 in the Supporting Information), although the morphological characterization indicates significant coalescence of nanoparticles at that temperature, suggesting that surface-localized probe environments remain dominant through this stage of thermal evolution.

The spectrum measured at 800 °C exhibits the most significant modification of the hyperfine response (Figure 6e), exhibiting substantially modified spectral modulation compared to lower temperatures. At this temperature, three distinct environments are detected. In addition to Site 1 that retains a broad frequency distribution, as observed at lower temperatures, two well-defined interactions emerge. Sites 2 and 3 exhibit hyperfine parameters characteristic of probes in more ordered environments, with null or near-null asymmetry parameters and narrow frequency distributions (10% and 0%, respectively), and well-defined frequencies (ν_*Q*2_ ≈ 404 MHz and ν_*Q*3_ ≈ 12 MHz, respectively). The appearance of these new hyperfine environments is fully consistent with the structural transformation observed by XRD at 800 °C, which reveals the coexistence of two distinct crystalline phases, MoO_2_ and β-Mo_2_C, after partial reduction of the oxide phase.

Nevertheless, we explicitly acknowledge that the unambiguous assignment of sites 2 and 3 at 800 °C to specific crystalline phases (MoO_2_ or β-Mo_2_C) is not yet possible based on the currently available data and remains uncertain. To the best of our knowledge, systematic hyperfine studies of ^111^In (^111^Cd) in MoO_2_ and β-Mo_2_C are currently unavailable in the literature, preventing a definitive identification of the observed frequencies. Recent TDPAC investigations of Cd impurities in MoO_3_ have demonstrated that oxygen-vacancy configurations and local lattice distortions can generate significantly different electric field gradients around the probe nuclei.^51^ Therefore, it is plausible that the interactions observed at 800 °C reflect a combination of distinct local environments associated with MoO_2_, β-Mo_2_C, oxygen-deficient regions, and interface-related configurations. However, additional experimental and theoretical investigations, particularly first-principles EFG calculations, will be required before definitive crystallographic assignments can be established.

Overall, the main contribution of the TDPAC measurements is not the definitive identification of all crystallographic probe sites, but rather the direct observation of local structural evolution throughout the decomposition pathway. The results reveal the transition from highly disordered environments to locally ordered regions, the onset of crystallization prior to the development of long-range order detectable by XRD, the strong influence of nanoparticle surfaces and interfaces during growth, and the emergence of new local environments associated with the formation of the MoO_2_/β-Mo_2_C composite system at high temperatures. Specifically, the TDPAC measurements make three significant contributions to this study: first, the detection of the early stages of local crystalline ordering at 400 °C, providing direct evidence of MoO_2_ nucleation before it can be detected by XRD; second, the tracking of the migration of probes from interfacial/grain-boundary environments to nanoparticle surface environments as the system evolves between 500 and 600 °C; and third, the identification of multiple distinct local environments at 800 °C, indicating a hybrid MoO_2_/β-Mo_2_C system. However, the exact crystallographic assignment of each hyperfine site still needs to be determined. These results demonstrate the atomic-scale sensitivity of TDPAC to structural transitions and local disorder, which cannot be observed by XRD alone. On the whole, this provides a comprehensive understanding of the crystallization and reduction pathway of molybdenum­(II) acetate at different temperatures.

Figure 7 summarizes the evolution of the hyperfine parameters as a function of temperature, together with a schematic illustration of the formation process and the probable locations of the ^111^In­(^111^Cd) probe nuclei at each stage of morphological evolution according to the discussion previously presented. The progressive decrease in the asymmetry parameter η with increasing temperature is consistent with a gradual improvement in local structural ordering and a reduction in defect density, in qualitative agreement with the macroscopic increase in crystallinity observed by XRD across the same temperature range. This correlation reinforces the self-consistent picture emerging from the combined multitechnique analysis: local atomic ordering, as probed by TDPAC, and long-range crystalline order, as probed by XRD, evolve in a correlated manner during the thermal decomposition of the precursor, with the local ordering consistently preceding the macroscopic crystallization detectable by diffraction methods.

**Figure 7:**
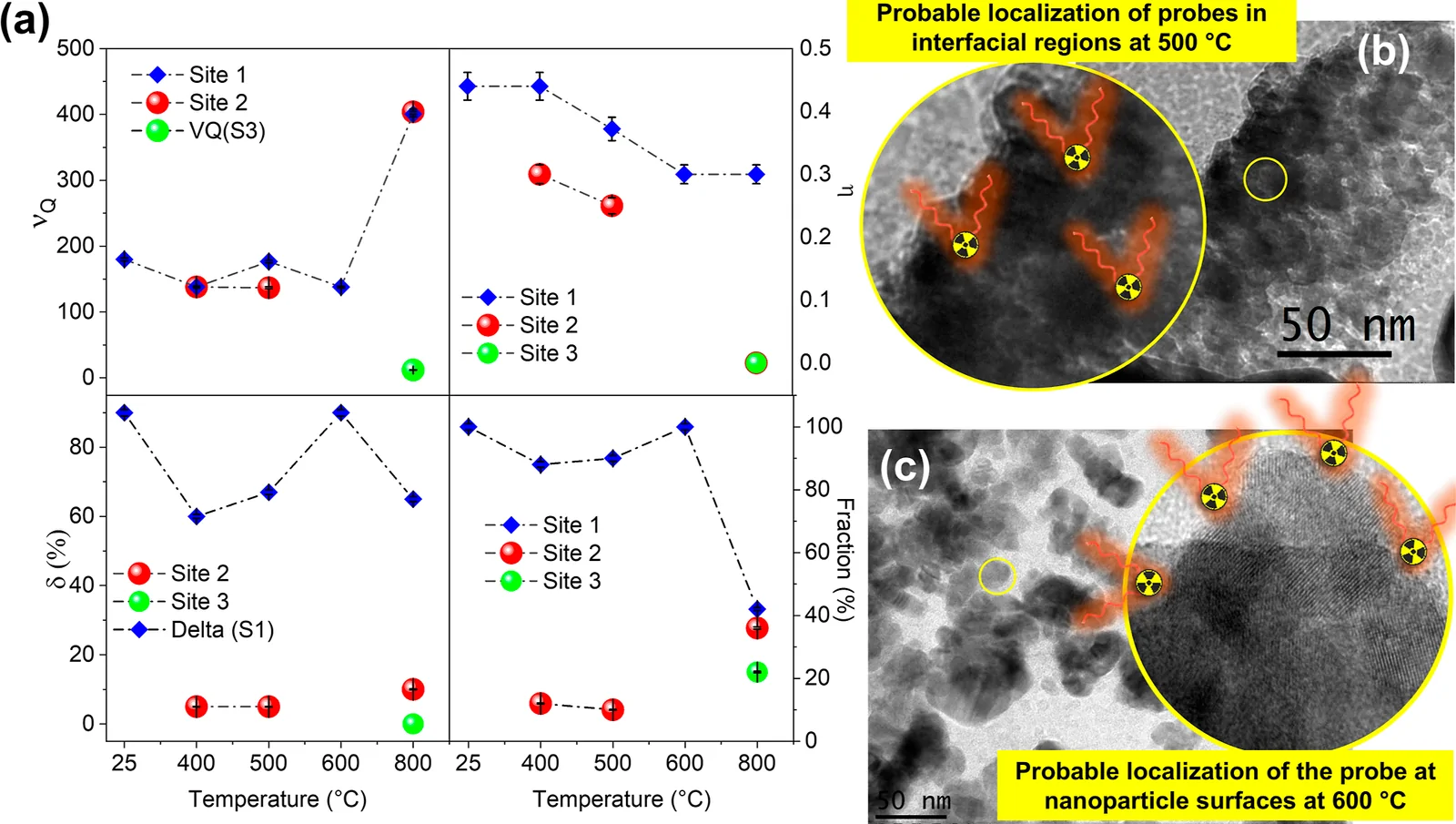
Hyperfine parameters as a function of temperature and a schematic representation of the probable locations of the probe nuclei at 500 and 600 °C: (a) variation of hyperfine parameters in the range from 25 to 800 °C; (b) probable location of the probe in interfacial regions at 500 °C; and (c) probable location of the probe in surface regions of the nanoparticles at 600 °C.

Taken together, the XRD, SEM/HRTEM, thermal analysis, and TDPAC results provide a coherent description of the transformation pathway. The decomposition of molybdenum­(II) acetate first generates an amorphous matrix containing locally ordered regions, followed by the nucleation of MoO_2_ nanodomains, growth into crystalline nanoparticles, and eventual coalescence into larger structures. At the highest temperature investigated, oxygen loss and carbon incorporation induce partial conversion to β-Mo_2_C, producing a hybrid oxide–carbide system. The excellent agreement between local and long-range probes strengthens the proposed mechanistic model.

We emphasize that the combined results suggest a three-stage transformation pathway: (i) degradation and amorphization of the precursor between 300 and 400 °C, accompanied by the emergence of locally ordered nuclei that can only be detectable only by TDPAC; (ii) nucleation and crystallization of MoO_2_ nanoparticles between 500 and 700 °C, characterized by increasing long-range order and strong contributions from surface and interfacial environments; and (iii) partial reduction of MoO_2_ and formation of the MoO_2_/β-Mo_2_C hybrid system at 800 °C, generating new crystalline local environments associated with the coexistence of metallic and oxide phases (see Table S2 in the Supporting Information). This correlation highlights the complementary nature of TDPAC and XRD, demonstrating that local atomic ordering precedes the development of long-range crystallinity during the thermal decomposition process.

Finally, Figure 8 schematically summarizes the thermal decomposition pathway of molybdenum­(II) acetate (Mo­(CH_3_COO)_2_) up to the formation of MoO_2_ and a small fraction of β-Mo_2_C. Additionally, Figure S5 in the Supporting Information provides a schematic summary of the structural and microstructural correlations established throughout this study, integrating the key transformation stages, including precursor amorphization, nanoparticle nucleation, progressive crystallization of MoO_2_, and partial carburization leading to the formation of the hybrid MoO_2_/β-Mo_2_C system across the investigated temperature range. This diagram synthesizes the complementary insights obtained from XRD, SEM/HRTEM, thermal analysis, and hyperfine interaction measurements, illustrating how defect-mediated processes govern the sequential evolution of the Mo-based system during thermal treatment. Overall, the multimodal experimental approach employed here provides a comprehensive mechanistic understanding of the thermal decomposition of molybdenum­(II) acetate, revealing how local atomic environments, defects, and structural heterogeneities govern phase evolution and microstructural development throughout the transformation process. These findings provide valuable insights into the mechanisms controlling crystallization, reduction, and carburization in molybdenum-based materials.

**Figure 8:**
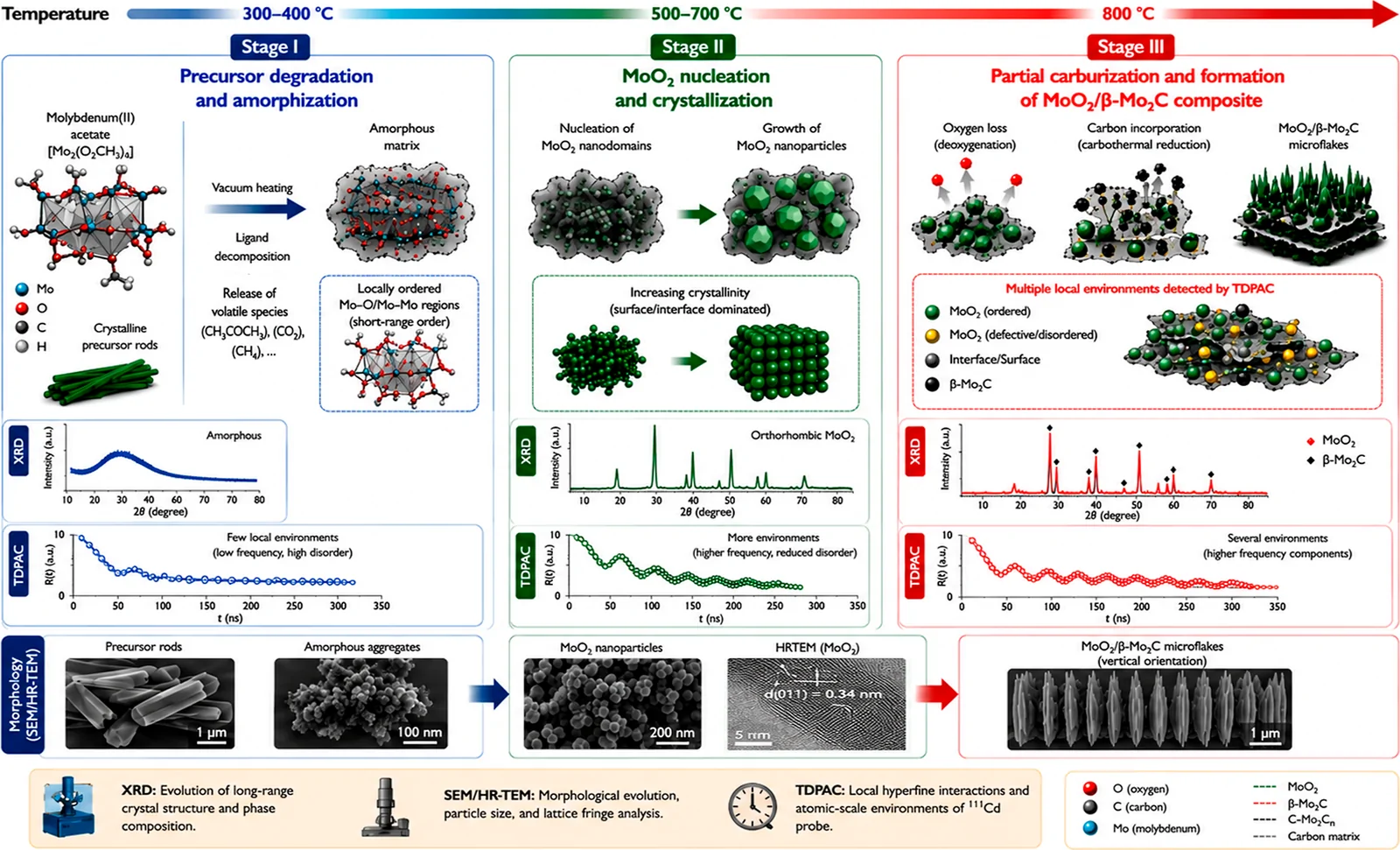
Schematic summary of the thermal decomposition pathway of molybdenum­(II) acetate under vacuum and the corresponding structural, morphological, and atomic-scale evolution revealed by complementary characterization techniques. Stage I (300 °C–400 °C) involves precursor degradation, ligand decomposition, and amorphization, resulting in the formation of a structurally disordered matrix containing short-range ordered Mo–O/Mo–Mo regions. Although XRD indicates predominantly amorphous behavior, TDPAC detects the emergence of locally ordered environments, evidencing the onset of nucleation processes. Stage II (500 °C–700 °C) is characterized by the nucleation, growth, and crystallization of monoclinic MoO_2_ nanoparticles, accompanied by increasing long-range order and strong contributions from surface and interfacial environments due to the nanoscale dimensions of the particles. Stage III (800 °C) involves partial carbothermal reduction of MoO_2_, promoted by carbonaceous species generated during acetate decomposition, leading to the formation of a hybrid MoO_2_/β-Mo_2_C system composed of microflake-like structures decorated with nanoparticles. The lower panels summarize the evolution observed by XRD, TDPAC, and SEM/HRTEM, highlighting the correlation between local atomic ordering, crystallization, nanoparticle growth, and phase transformation throughout the thermal treatment. The diagram illustrates how local ordering detected by TDPAC precedes the development of long-range crystallinity observed by XRD and provides an integrated mechanistic description of the formation of the MoO_2_/β-Mo_2_C composite system.

### 2.4 Conclusions

The study presented here establishes molybdenum­(II) acetate as an efficient and versatile solid precursor for the controlled synthesis of Mo-based nanostructures. Thermal decomposition under vacuum proceeds through a well-defined three-stage pathway: precursor degradation, amorphization, and nucleation between 300 and 400 °C; growth and crystallization of phase-pure monoclinic MoO_2_ nanoparticles between 500 and 700 °C; and partial carbothermal reduction at 800 °C, leading to the formation of mixed MoO_2_/β-Mo_2_C microstructures.

Structural and morphological analyses demonstrate that the MoO_2_ phase forms as nanoparticles with an average size of 23 ± 7.9 nm, exhibiting high thermal stability up to 600 °C. At higher temperatures, increased atomic mobility promotes particle coalescence and phase transformation, resulting in hybrid MoO_2_/β-Mo_2_C systems characterized by the emergence of vertically oriented microflakes decorated with nanoparticles.

Crucially, TDPAC spectroscopy provides atomic-scale confirmation of these transformations, enabling the direct identification of the amorphous and crystalline MoO_2_ environments. The evolution of the quadrupole frequency, asymmetry parameter, and frequency distribution reveals progressive local ordering, defect rearrangement, and surface-to-bulk transitions that are not accessible by using conventional techniques alone.

The present approach offers several advantages, including the use of a single-source organometallic precursor, self-reducing decomposition conditions generated by acetate ligands, the formation of crystalline MoO_2_ without external reducing gases, relatively simple vacuum-sealed thermal processing, and access to both oxide and carbide phases through temperature control.

Despite the comprehensive characterization presented here, some limitations remain. In particular, the exact crystallographic assignment of the hyperfine interactions observed by TDPAC could not be established due to the absence of reference data for ^111^Cd in MoO_2_ and β-Mo_2_C. Future studies combining density functional theory calculations of electric field gradients with complementary spectroscopic techniques such as XPS, Raman spectroscopy, or X-ray absorption spectroscopy could provide a more detailed understanding of defect structures, oxygen vacancies, and local chemical environments.

The synthesis strategy demonstrated in this work opens several possibilities for future research. The ability to tune the relative fractions of MoO_2_ and β-Mo_2_C through controlled thermal processing may enable the design of oxide–carbide heterostructures optimized for electrocatalysis, hydrogen evolution reactions, energy-storage electrodes, and electronic applications. Furthermore, the methodology developed here may be extended to other transition-metal carboxylate precursors, providing a general framework for understanding decomposition-driven nanostructure formation through combined structural and atomic-scale characterization.

## 3 Experimental Section

### 3.1 Synthesis and Characterization of Samples

The precursor material used in this study was molybdenum­(II) acetate, [Mo_2_(O_2_CH_3_)_4_], Merck 98%. For the thermal decomposition of the precursor, approximately 80 mg of the compound was inserted into quartz tubes, evacuated for 1 h (approximately ∼10^–3^ mbar), vacuum-sealed, and subsequently subjected to heat treatments between 300 and 800 °C for 6 h.

To perform time-dependent perturbed angular correlation spectroscopy (TDPAC) measurements, it is necessary to introduce radioactive probe nuclei into the structure of the material to be studied. In this work, the well-known ^111^Cd probe nucleus was used, which is frequently employed due to its excellent nuclear characteristics. The radioactive parent nucleus ^111^In decays by electron capture, forming ^111^Cd. The data obtained by TDPAC provide the time-dependent anisotropy ratio function, given by $R\left(t\right)=A_{22}\sum_{i}f_{i}G_{22}^{i}\left(t\right)$ , where *f*_*i*_ represents the fraction of probe nuclei in different environments and *G*_22_(*t*) is the corresponding perturbation function. The angular correlation coefficient *A*_22_, for the system ^111^In­(^111^Cd), is equal to −0,18, implying that the function *R*(*t*) must be represented with the *y* axis inverted. Details on the TDPAC fitting of spectra are given by Silva et al.^67^ and on the TDPAC technique can be found elsewhere.^47,68,69^

In the case of electric quadrupole hyperfine interactions, the perturbation factor is given by $G_{22}\left(t\right)=\sum_{n=0}^{n_{\max}}S_{kn}\;\cos\left(n\omega_{0}t\right)$ , in which the frequency ω_0_ is defined by ω_0_ = *g*_*n*_(η)­ν_*Q*_, with ν_*Q*_ = *eQV*_*zz*_/*h* being the quadrupole frequency. The frequency ω_0_ and the coefficients *S*_*kn*_ depend on the asymmetry parameter η, defined as η = (*V*_*xx*_ – *V*_*yy*_)/*V*_*zz*_. In TDPAC measurements, it is then possible to extract the values of η and ν_*Q*_, allowing the calculation of the components *V*_*kk*_, *k* = *x*, *y*, *z*, of the electric field gradient tensor. The parameter η quantifies the deviation from the axial symmetry of the local charge distribution, while *V*_*zz*_ provides information about the charge density around the probe nuclei. More information on TDPAC in nanomaterials is found elsewhere.^48–50,70^

For TDPAC experiments, pellets of the organometallic precursor were prepared using a hydraulic press, applying a pressure of 4 tons. A few drops of ^111^InCl_3_ solution were deposited onto these pellets, which were subsequently vacuum-dried and subjected to heat treatment at 300 °C for 6 h to diffuse the probe nucleus into the material matrix. The samples were then classified as radioactive and nonradioactive according to the purpose of the characterization. Apart from the incorporation of the ^111^In radioactive probe required for TDPAC measurements, both sample sets were prepared from the same precursor and subsequently subjected to identical thermal-treatment protocols, including the same annealing temperatures, treatment durations, vacuum-sealed quartz tube conditions, and cooling procedures. Therefore, the structural, morphological, and hyperfine results discussed throughout this work were obtained from samples with equivalent thermal histories.

The set of nonradioactive samples was characterized using conventional materials characterization techniques. Crystal structures were determined by X-ray diffraction (XRD) using a Rigaku SmartLab SE diffractometer equipped with Cu Kα radiation (λ = 1.5406 Å). The diffraction patterns were analyzed by Rietveld refinement using the Profex graphical interface coupled to the BGMN refinement engine. Successive refinement cycles were performed to determine phase fractions, crystallographic parameters, and structural information associated with the thermal evolution of the samples. In addition, the relative crystallinity of the samples was estimated using the relative crystallinity equation based on the integrated intensity of the crystalline diffraction peaks.^71^ Detailed Rietveld refinement results, including refined lattice parameters, phase fractions and refinement quality indicators, are provided in Table S1 of the Supporting Information. Morphology and elemental composition were investigated by field-emission scanning electron microscopy (FEG-SEM, JEOL JSM-6701F) equipped with an energy-dispersive X-ray spectroscopy (EDS) detector. SEM images were used to evaluate the overall morphology of the samples and to determine the dimensional distributions of the microflake structures formed at 800 °C, including their length, width, and thickness. High-resolution transmission electron microscopy (HRTEM, JEOL JEM-2100) was employed to investigate the local crystal structure of the synthesized materials through measurements of interplanar spacings and analysis of fast Fourier transform (FFT) patterns obtained from selected HRTEM images. The FFT patterns were interpreted as virtual electron diffraction patterns and used to support phase identification and structural characterization. HRTEM images were also used to determine the particle size distribution of the MoO_2_ nanoparticles obtained at 600 °C. Quantitative size analyses were performed using multiple SEM and HRTEM micrographs acquired from different regions of each sample to ensure representative statistical sampling. Image measurements were carried out using ImageJ software, and the resulting data sets were statistically analyzed using Python-based scientific libraries. Thermal behavior was investigated by simultaneous thermogravimetric and differential scanning calorimetry analyses (TGA/DSC, TA Instruments SDT Q600) performed from 30 to 900 °C at a heating rate of 10 °C min^–1^ under a nitrogen flow of 100 mL min^–1^. Thermal analyses were carried out for both the molybdenum­(II) acetate precursor and the MoO_2_ sample obtained after thermal treatment at 500 °C, aiming to evaluate the thermal evolution of the precursor and the stability of the resulting oxide phase.

The set of radioactive samples was characterized by TDPAC. The previously vacuum-sealed quartz tubes containing the radioactive samples were placed in a furnace and subjected to measurements between 25 and 800 °C, with an approximate duration of 6 h at each temperature, during which the *R*(*t*) functions were acquired. All TDPAC measurements were performed at the Hyperfine Interactions Laboratory (LIH) of the Research Reactor Center (CERPQ/IPEN). Figure 9 presents a systematic description of the procedures adopted for both sample groups.

**Figure 9:**
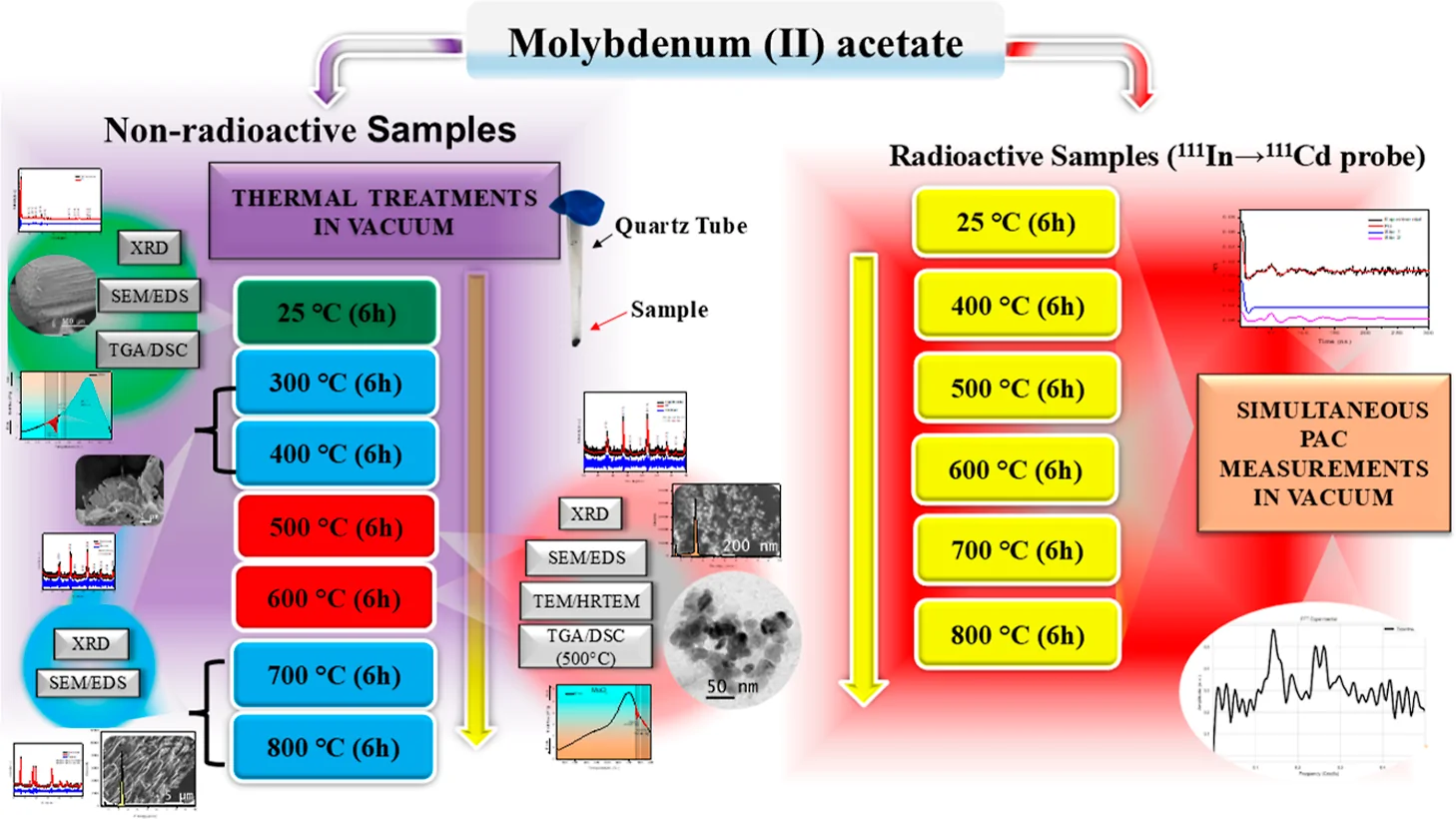
Systematization of characterization techniques used in each sample set (radioactive): TDPAC and (nonradioactive): XRD, SEM/HRTEM, and thermal analyses (TGA/DSC).

## Supplementary Material



## Notes

Thermal analyses (DSC, TGA/DTG) and X-ray diffraction of molybdenum­(II) acetate and the MoO_2_ sample obtained at 500 °C (Figure S1); comparison between experimental, fitted, and theoretical XRD patterns for the sample treated at 800 °C, showing the formation of β-Mo_2_C (Figure S2); statistics on MoO_2_ nanoparticle diameters at 600 °C obtained via TEM analysis (Figure S3); TDPAC spectrum obtained at 700 °C (Figure S4); schematic summary of the correlation between amorphization, nucleation, crystallization, and carburization processes (Figure S5); structural evolution and lattice parameters of molybdenum acetate during heat treatment (Table S1); hyperfine parameters obtained from TDPAC measurements as a function of temperature (Table S2); and relative fractions of crystalline and amorphous phases obtained from XRD patterns (Table S3) (PDF)
