# Supplementary material for: Thermal Decomposition of Molybdenum(II) Acetate as a Controlled Route to MoO2 and Mo2C Nanostructures: Structural, Morphological, and Atomic-Scale Insights
Source: ACS Omega. 2026 Aug 17;11(34):51371–87. doi: 10.1021/acsomega.6c04468 (PMC13625115; doi:10.1021/acsomega.6c04468)
Supplement: Supplementary file 1 [file ao6c04468_si_001.pdf]

Thermal Decomposition of Molybdenum (II) Acetate as a Controlled Route to MoO_2 and Mo_2C Nanostructures: Structural, Morphological, and Atomic-Scale Insights

Katiusse Soares de Souza,[†] Crystian W. C. Silva,[†] Anastassia Burimova,[†] Levy Scalise,[†] Gabriel Cabrera-Pasca,[‡] Juliana Schell,[¶,§] Larissa Otubo,[†] and Artur Wilson Carbonari[*,†,‖]

†*Instituto de Pesquisas Energéticas e Nucleares, IPEN-CNEN/SP, 05508-000, São Paulo, SP, Brazil*

‡*Universidade Federal do Pará (UFPA), Abaetetuba – Pará, 09210-580, Brazil*

¶*European Organization for Nuclear Research (CERN), CH-1211, Geneva, Switzerland*

§*Institute for Materials Science and Center for Nanointegration Duisburg-Essen (CENIDE), University of Duisburg-Essen, 45141, Essen, Germany*

‖*European Organization for Nuclear Research (CERN), Esplanade des Particules 1, 1217 Meyrin, Switzerland*

E-mail: carbonar@ipen.br

1 Suplementary Information

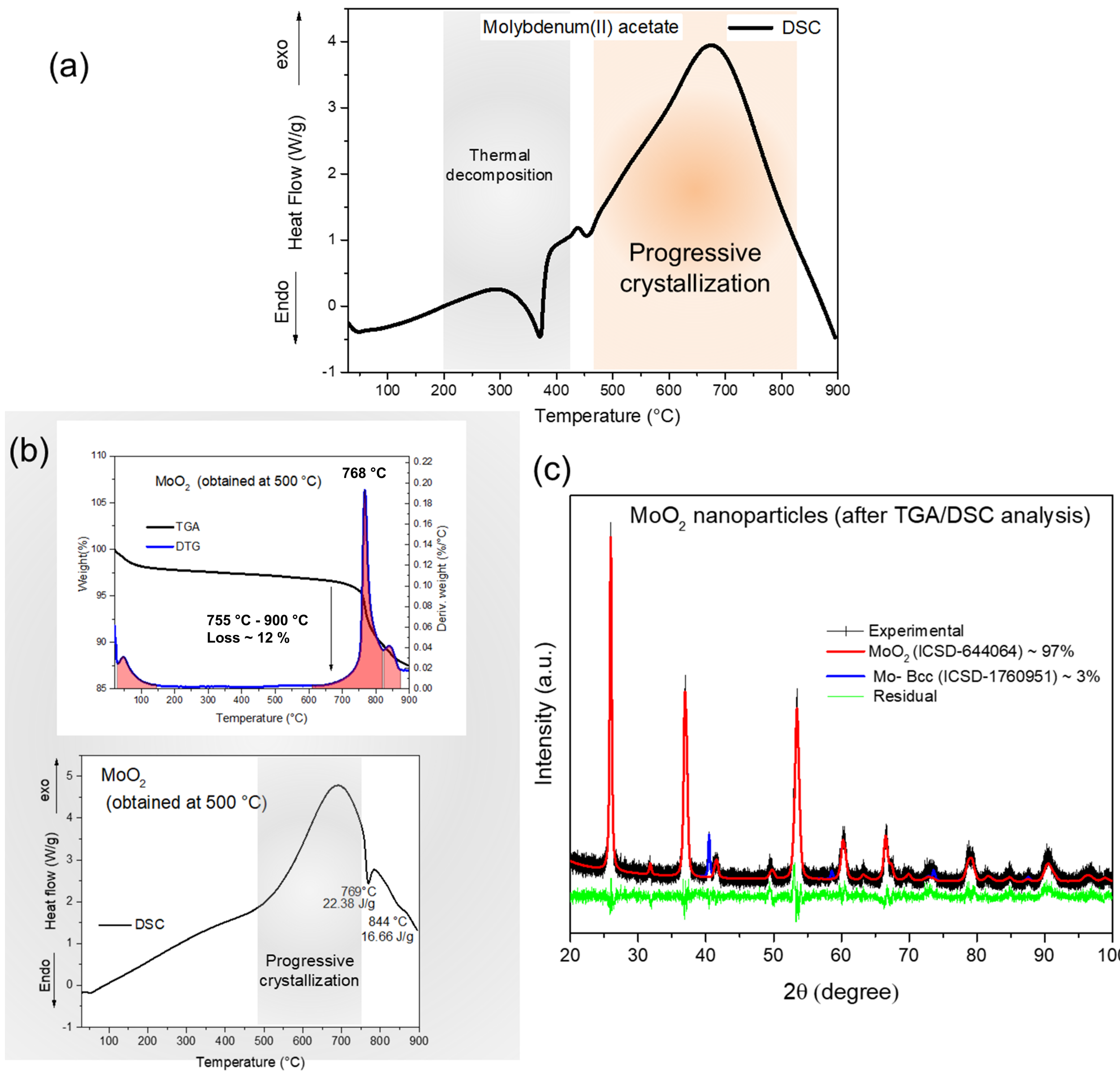

Figure S1: Thermal analysis of molybdenum(II) acetate and the MoO_2 sample prepared at 500 °C, both performed under a nitrogen atmosphere. (a) DSC curve of molybdenum(II) acetate, highlighting the precursor decomposition region (300–400 °C) and the progressive crystallization process consistent with the increase in MoO_2 crystallinity between 500 and 700 °C. (b) TGA/DTG and DSC curves of the MoO_2 sample prepared at 500 °C, showing a mass-loss event centered at approximately 769 °C. (c) Rietveld refinement of the X-ray diffraction pattern collected after the TGA/DSC experiment, revealing predominantly monoclinic MoO_2 (97 wt%, ICSD 644064) together with a minor Mo-BCC phase (3 wt%, ICSD 1760951), consistent with oxygen loss at elevated temperatures.

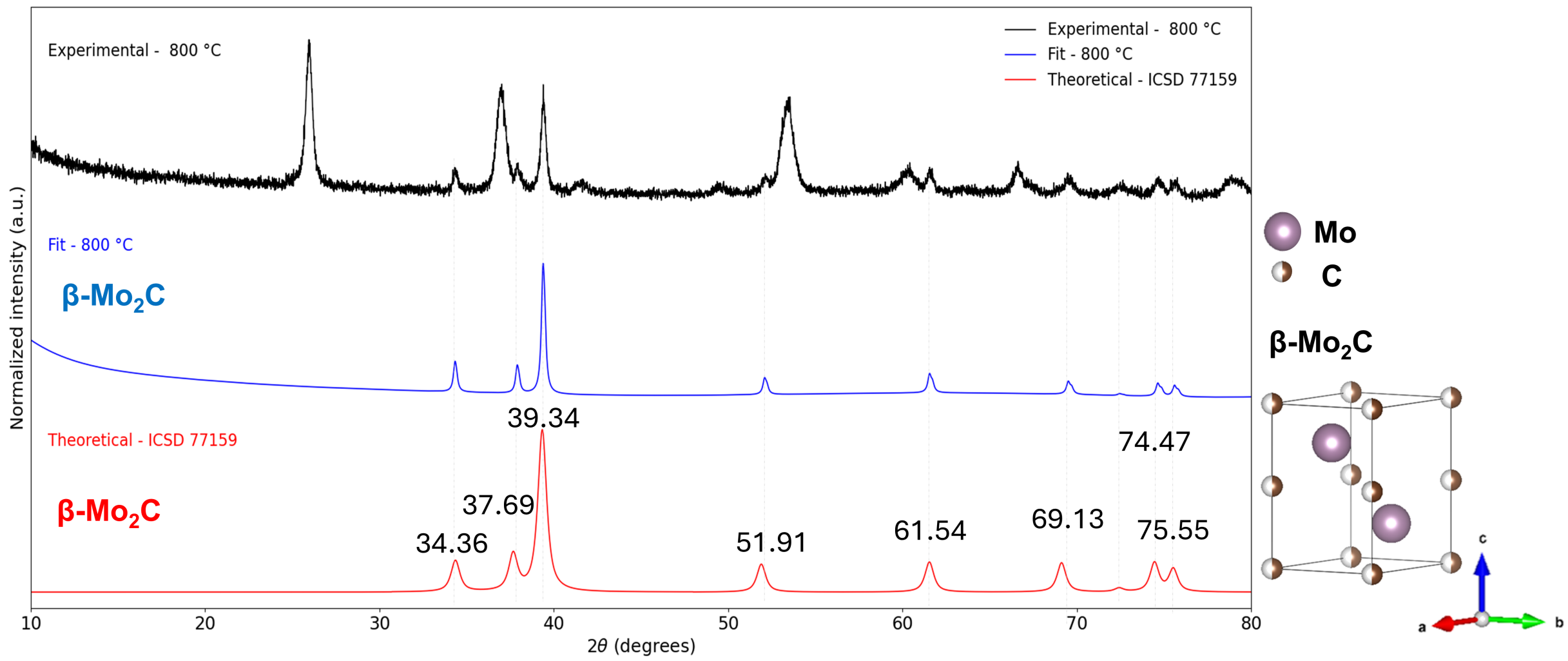

Figure S2: Comparison between the experimental XRD pattern collected for the sample treated at 800 °C, the corresponding fitted profile, and the theoretical diffraction pattern of hexagonal β-Mo_2C (ICSD 77159). The main diffraction peaks associated with the β-Mo_2C phase are indicated, highlighting the formation of molybdenum carbide during the high-temperature thermal decomposition of molybdenum(II) acetate under vacuum. The crystal structure of β-Mo_2C is shown on the right for reference.

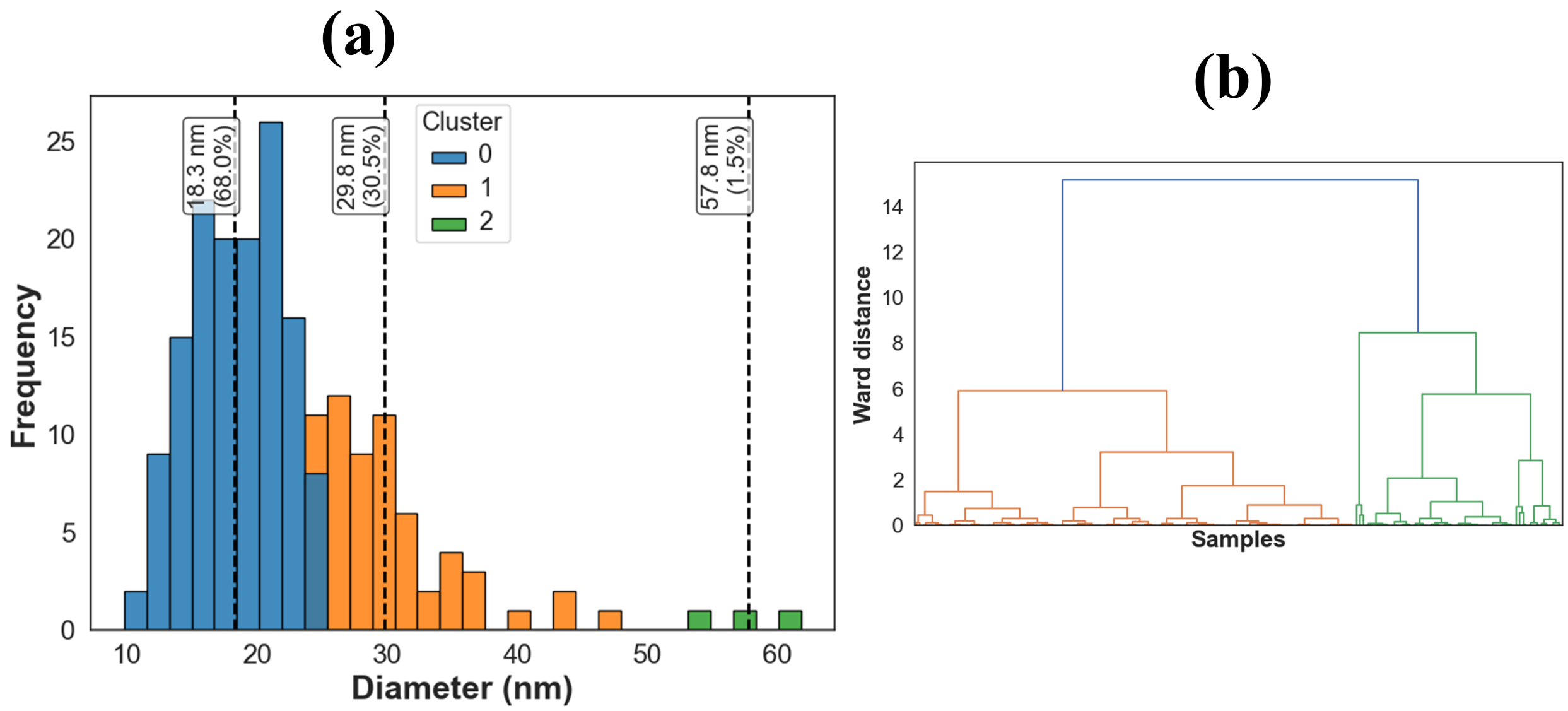

Figure S3: Descriptive statistics of the nanoparticle diameters of MoO2 at 600 °C, obtained from TEM analyses. (a) Histogram of the diameter distribution. (b) Hierarchical clustering.

.

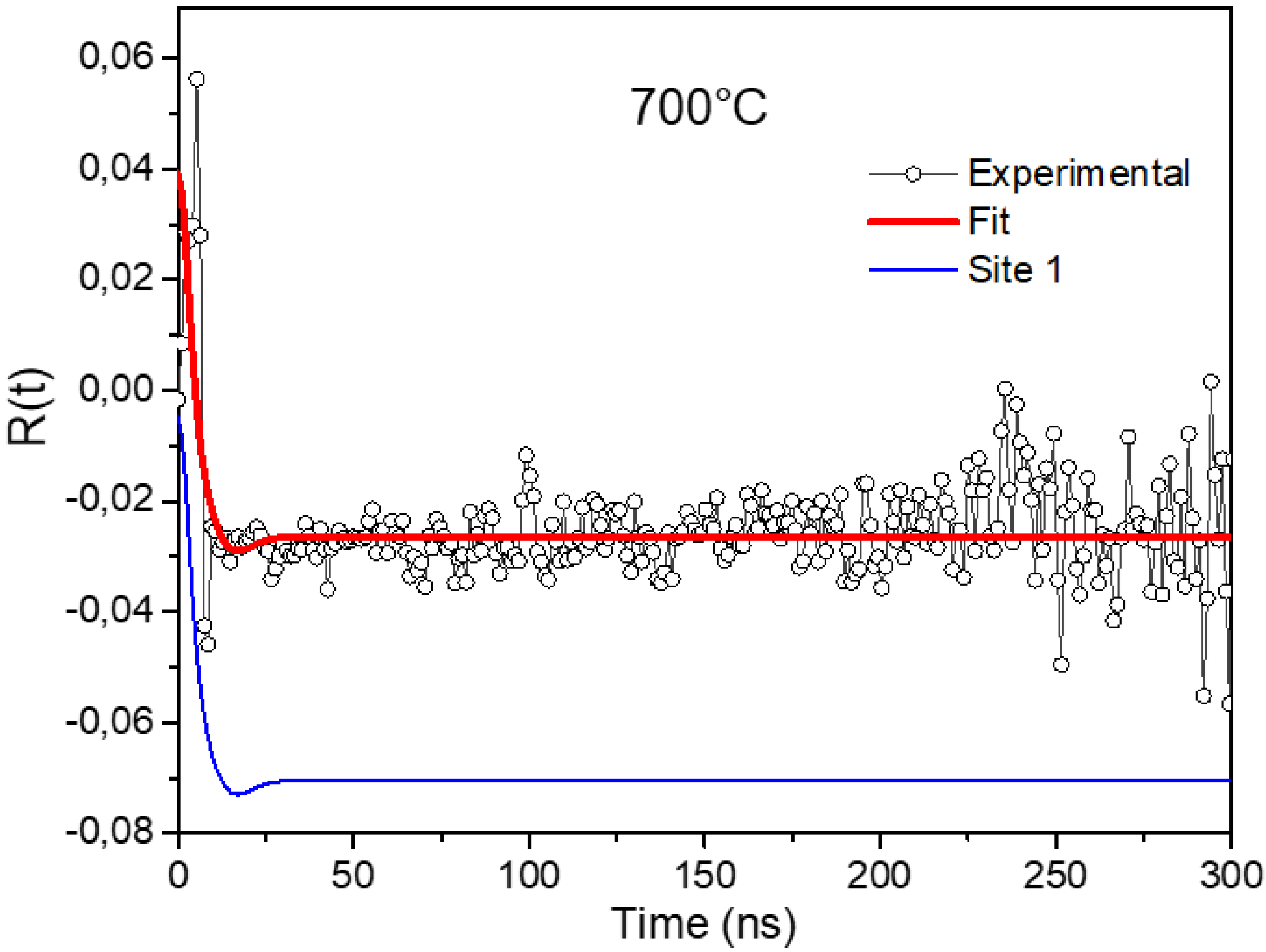

Figure S4: Time-dependent anisotropy ratio function TDPAC obtained at 700 °C.

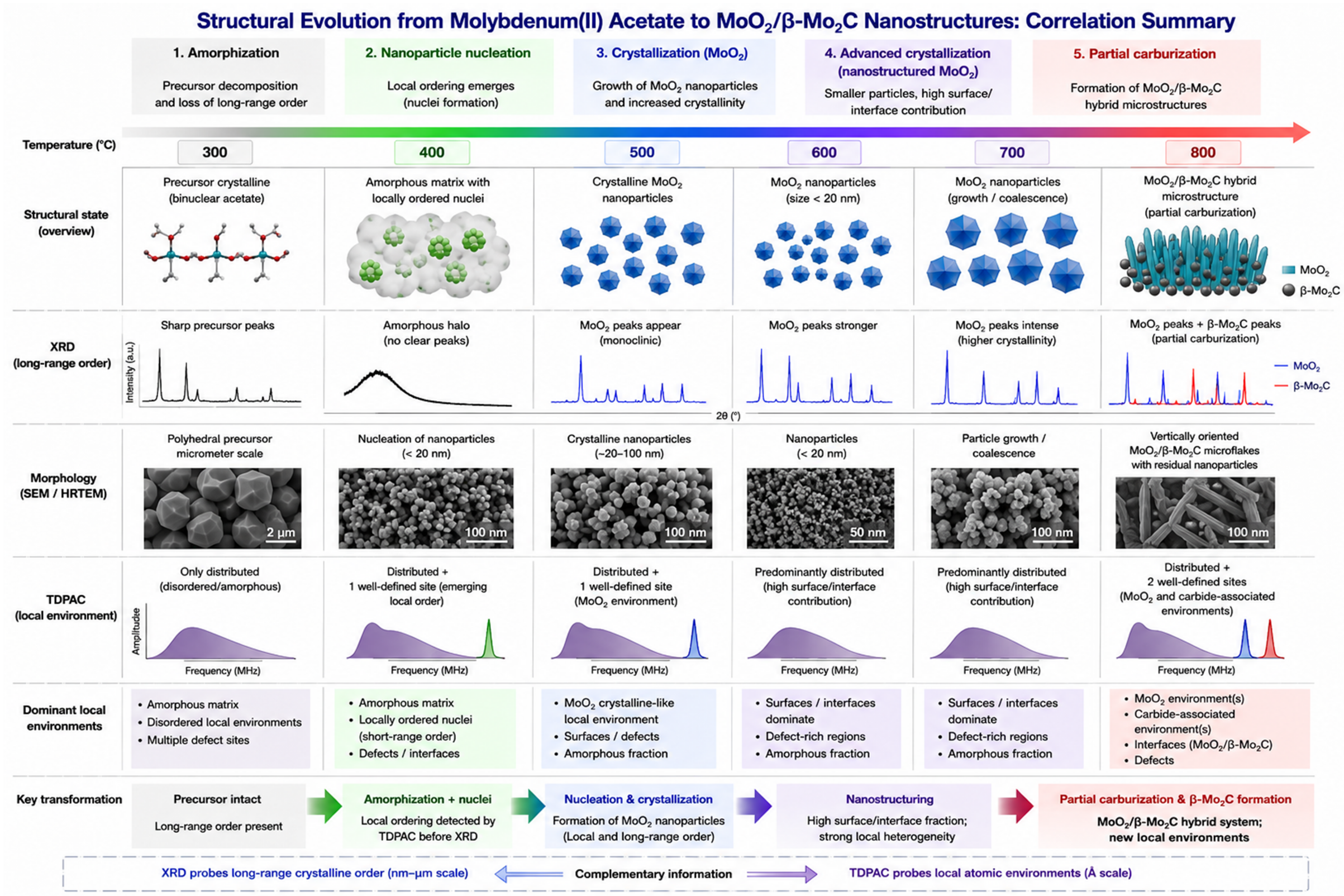

Figure S5: Schematic correlation summary linking amorphization, nanoparticle nucleation, crystallization of MoO_2, and partial carburization leading to the formation of hybrid MoO_2/β-Mo_2C microstructures across the investigated temperature range.

Table S1: Structural evolution of molybdenum acetate during thermal treatment under vacuum. Experimental lattice parameters obtained from Rietveld refinement.

Temperature	Phase	Crystal system	Experimental lattice parameters (Å)	Phase fraction (%)	χ^2	ICSD
RT	Molybdenum acetate	Triclinic	$a = 8.41$, $b = 5.50$, $c = 7.52$	100	1.01	1213070
300 °C	Molybdenum acetate	Triclinic	$a = 8.41$, $b = 5.50$, $c = 7.51$	100	1.03	1213070
400 °C	Amorphous	–	–	100	–	–
500 °C	MoO_2	Monoclinic	$a = 5.51$, $b = 4.86$, $c = 5.61$	100	1.05	644064
600 °C	MoO_2	Monoclinic	$a = 5.57$, $b = 4.81$, $c = 5.69$	100	1.20	644064
700 °C	MoO_2	Monoclinic	$a = 5.57$, $b = 4.85$, $c = 5.61$	100	1.03	644064
800 °C	MoO_2	Monoclinic	$a = 5.59$, $b = 4.82$, $c = 5.61$	83.52	1.01	644064
800 °C	β-Mo_2C	Hexagonal	$a = 3.00$, $c = 4.73$	16.48	1.01	77159

Table S2: Hyperfine parameters obtained from TDPAC measurements as a function of temperature. Relative uncertainties of 1% for the site fractions, ν_Q, and δ, and 5% for η were assumed.

**Temperature (°C)**	**Fraction (%)**	$\boldsymbol{\nu_Q}$ (MHz)	$\boldsymbol{\eta}$	$\boldsymbol{\delta}$ (%)
		**Site 1**		
25	100 ± 1.00	180 ± 1.80	0.44 ± 0.022	90 ± 0.90
400	88 ± 0.88	138.184 ± 1.382	0.44 ± 0.022	60 ± 0.60
500	90 ± 0.90	176.623 ± 1.766	0.3722 ± 0.0186	67 ± 0.67
600	100 ± 1.00	138 ± 1.38	0.30 ± 0.015	90 ± 0.90
800	42 ± 0.42	400.574 ± 4.006	0.30 ± 0.015	65 ± 0.65
		**Site 2**		
25	–	–	–	–
400	12 ± 0.12	138 ± 1.38	0.30 ± 0.015	5 ± 0.05
500	10 ± 0.10	138 ± 1.38	0.25 ± 0.0125	5 ± 0.05
600	–	–	–	–
800	36 ± 0.36	404 ± 4.04	0 ± 0	10 ± 0.10
		**Site 3**		
25	–	–	–	–
400	–	–	–	–
500	–	–	–	–
600	–	–	–	–
800	22 ± 0.22	12 ± 0.12	0 ± 0	0 ± 0

2 Crystalline and amorphous relative content from XRD diffractograms

To estimate the crystalline and amorphous content from an X-ray Diffraction (XRD) pattern, you can use the Integration Method (also known as the Ruland-Vonk method). This technique separates the sharp peaks (from the crystalline phase) from the broad background "humps" (from the amorphous phase).

The degree of crystallinity is calculated using the following equation:

$$\text{Crystallinity}(\%) = \left(\frac{A_{\text{crystalline}}}{A_{\text{crystalline}} + A_{\text{amorphous}}} \right) \times 100 \tag{1}$$

where $A_{\text{crystalline}}$ is the total integrated area of all sharp crystalline peaks, $A_{\text{amorphous}}$ is the

total integrated area of the broad background halos attributed to the amorphous phase, and ($A_{\text{crystalline}} + A_{\text{amorphous}}$) represents the total area under the entire XRD curve.

The formal framework for estimating crystallinity and amorphous fractions via wide-angle X-ray scattering (WAXS/XRD) relies on foundational papers published by Wilhelm Ruland (who derived the underlying physical theory)[https://doi.org/10.1107/S0365110X61003429, https://doi.org/10.1016/0032-3861(64)90123-5] and Cornelis G. Vonk (who digitized and adapted the method for automated computer processing)[https://doi.org/10.1107/S0021889873008332].

For a modern critical review showing how robust the combined Ruland-Vonk method stands up against modern alternative XRD algorithms (like peak deconvolution or the Segal method), see the paper of M. Ingo et al.[https://doi.org/10.1007/s10570-023-05127-6]
